# Association of Accelerometer-Measured Physical Activity Level With Risks of Hospitalization for 25 Common Health Conditions in UK Adults

**DOI:** 10.1001/jamanetworkopen.2022.56186

**Published:** 2023-02-16

**Authors:** Eleanor L. Watts, Pedro F. Saint-Maurice, Aiden Doherty, Georgina K. Fensom, Joshua R. Freeman, Jessica S. Gorzelitz, David Jin, Kathleen M. McClain, Keren Papier, Shreya Patel, Eric J. Shiroma, Steven C. Moore, Charles E. Matthews

**Affiliations:** 1 Metabolic Epidemiology Branch, Division of Cancer Epidemiology and Genetics, National Cancer Institute, Rockville, Maryland; 2 Big Data Institute, Nuffield Department of Population Health, British Heart Foundation Centre of Research Excellence, University of Oxford, Oxford, United Kingdom; 3 Cancer Epidemiology Unit, Nuffield Department of Population Health, Oxford, United Kingdom; 4 Department of Health and Human Physiology, University of Iowa, Iowa City; 5 Laboratory of Epidemiology and Population Science, National Institute on Aging, Bethesda, Maryland

## Abstract

**Importance:**

Higher physical activity levels are associated with lower risks of cancer, cardiovascular disease, and diabetes, but associations with many common and less severe health conditions are not known. These conditions impose large health care burdens and reduce quality of life.

**Objectives:**

To investigate the association between accelerometer-measured physical activity and the subsequent risk of hospitalization for 25 common reasons for hospitalization and to estimate the proportion of these hospitalizations that might have been prevented if participants had higher levels of physical activity.

**Design, Setting, and Participants:**

This prospective cohort study used data from a subset of 81 717 UK Biobank participants aged 42 to 78 years. Participants wore an accelerometer for 1 week (between June 1, 2013, and December 23, 2015) and were followed up over a median (IQR) of 6.8 (6.2-7.3) years; follow-up for the current study ended in 2021 (exact date varied by location).

**Exposures:**

Mean total and intensity-specific accelerometer-measured physical activity.

**Main Outcomes and Measures:**

Hospitalization for the most common health conditions. Cox proportional hazards regression analysis was used to estimate hazard ratios (HRs) and 95% CIs for mean accelerometer-measured physical activity (per 1-SD increment) and risks of hospitalization for 25 conditions. Population-attributable risks were used to estimate the proportion of hospitalizations for each condition that might be prevented if participants increased their moderate to vigorous physical activity (MVPA) by 20 minutes per day.

**Results:**

Among 81 717 participants, the mean (SD) age at accelerometer assessment was 61.5 (7.9) years; 56.4% were female, and 97.0% self-identified as White. Higher levels of accelerometer-measured physical activity were associated with lower risks of hospitalization for 9 conditions: gallbladder disease (HR per 1 SD, 0.74; 95% CI, 0.69-0.79), urinary tract infections (HR per 1 SD, 0.76; 95% CI, 0.69-0.84), diabetes (HR per 1 SD, 0.79; 95% CI, 0.74-0.84), venous thromboembolism (HR per 1 SD, 0.82; 95% CI, 0.75-0.90), pneumonia (HR per 1 SD, 0.83; 95% CI, 0.77-0.89), ischemic stroke (HR per 1 SD, 0.85; 95% CI, 0.76-0.95), iron deficiency anemia (HR per 1 SD, 0.91; 95% CI, 0.84-0.98), diverticular disease (HR per 1 SD, 0.94; 95% CI, 0.90-0.99), and colon polyps (HR per 1 SD, 0.96; 95% CI, 0.94-0.99). Positive associations were observed between overall physical activity and carpal tunnel syndrome (HR per 1 SD, 1.28; 95% CI, 1.18-1.40), osteoarthritis (HR per 1 SD, 1.15; 95% CI, 1.10-1.19), and inguinal hernia (HR per 1 SD, 1.13; 95% CI, 1.07-1.19), which were primarily induced by light physical activity. Increasing MVPA by 20 minutes per day was associated with reductions in hospitalization ranging from 3.8% (95% CI, 1.8%-5.7%) for colon polyps to 23.0% (95% CI, 17.1%-28.9%) for diabetes.

**Conclusions and Relevance:**

In this cohort study of UK Biobank participants, those with higher physical activity levels had lower risks of hospitalization across a broad range of health conditions. These findings suggest that aiming to increase MVPA by 20 minutes per day may be a useful nonpharmaceutical intervention to reduce health care burdens and improve quality of life.

## Key Points

**Question:**

Is physical activity associated with the risks of hospitalization for common health conditions?

**Findings:**

In this cohort study using accelerometer data from 81 717 UK Biobank participants, higher levels of physical activity, particularly moderate to vigorous intensity activity, were associated with lower risks of hospitalization for 9 of the 25 most common reasons for hospitalization. The largest decreases in risk were for gallbladder disease, diabetes, and urinary tract infections.

**Meaning:**

These findings suggest that increasing moderate to vigorous physical activity by 20 minutes per day may be a useful nonpharmaceutical intervention to reduce hospital admissions for many common health conditions, which could lower hospital burdens and improve quality of life.

## Teaser

This cohort study uses accelerometer data from UK Biobank participants to examine the association between physical activity and the risks of hospitalization for 25 common conditions and to estimate the proportion of hospitalizations that may be preventable with higher activity levels.

## Introduction

Substantial evidence suggests that higher levels of physical activity are associated with lower risks of cancer, diabetes, and cardiovascular disease.^1,2,3,4^ However, for many common and often less severe conditions, the associations of physical activity with the risks of hospitalization are not well characterized. These conditions reduce quality of life, are often risk factors for more severe diseases, and impose large health care burdens.^5^

Accelerometers measure the frequency, duration, and intensity of physical activity and capture the totality of activity throughout the day, such as incidental and/or unstructured activity, which is an important contributor to total physical activity energy expenditure.^6,7,8^ To date, large-scale prospective analyses of physical activity and disease risk have been almost entirely based on self-reported physical activity, which primarily captures leisure-time activity of moderate to vigorous intensity and is more susceptible to measurement error and biases, leading to uncertainty regarding the association between physical activity and health outcomes.

Using data from 81 717 participants in the UK Biobank accelerometer study, we aimed to (1) investigate the role of accelerometer-measured physical activity in the risks of hospitalization for 25 common conditions, (2) examine associations when substituting 20 minutes per day of sedentary time for 20 minutes per day of light physical activity (LPA) or moderate to vigorous physical activity (MVPA), and (3) estimate the proportion of hospitalizations that could be prevented with increased physical activity.

## Methods

### UK Biobank

The UK Biobank is an ongoing prospective cohort study. In brief, all participants are registered with the UK National Health Service and live within 40 km of a UK Biobank assessment center.^9^ Approximately 9.2 million people were initially invited to participate. Overall, 502 625 participants aged 40 to 69 years consented to join the cohort and attended 1 of 22 assessment centers throughout England, Wales, and Scotland between March 13, 2006, and October 1, 2010, representing a participation rate of 5.5%. The UK Biobank study was approved by the North West Multi-centre Research Ethics Committee. Written informed consent was provided by each participant before the study. This study followed the Strengthening the Reporting of Observational Studies in Epidemiology (STROBE) reporting guideline for cohort studies.^10^

### Baseline Assessment

Participants provided information on a range of sociodemographic, lifestyle, and health-related factors via a self-completed touch-screen questionnaire and a computer-assisted personal interview. Height and weight were measured at the assessment center. Details of the self-reported physical activity assessment are available in the eMethods in Supplement 1.

### Accelerometer Assessment

Between June 1, 2013, and December 23, 2015, participants who provided a valid email address were selected at random to receive email invitations to wear a wrist-worn accelerometer (AX3; Axivity Ltd) for 7 days to measure physical activity levels. The response rate was 45%.^11^ Participant characteristics were collected for a mean (SD) of 5.7 (1.1) years before accelerometer measurement. Follow-up for the current study ended on February 28, 2018, in Wales; July 31, 2020, in Scotland; and September 30, 2021, in England.

The total volume of physical activity was defined as the mean vector magnitude (in milligravity units).^11^ This approach has been externally validated against the doubly labeled water method.^12^ Time spent in sedentary activity (eg, driving or watching television), LPA (eg, cooking or self-care), MVPA (eg, walking the dog or jogging), and sleep were estimated using machine learning models that were trained using wearable cameras and time-use diaries among 152 individuals in free-living conditions.^13^

Overall, 103 696 participants had accelerometer data available We excluded 8041 respondents with incomplete or implausible accelerometer data (criteria used to determine whether data were implausible are provided in eFigure 1 in Supplement 1): 21 withdrew consent, 2961 had missing covariate data or were unable to walk (measured at baseline), 7520 had prevalent cancer (excluding nonmelanoma skin cancer [*International Statistical Classification of Diseases and Related Health Problems, Tenth Revision*, diagnostic code C44]), and 3439 had prevalent diabetes. Previous diagnosis of the conditions named may have altered their physical activity levels and risks of other diseases^14,15^ (eFigure 1 in Supplement 1). Our final analytic cohort included 81 717 participants aged 42 to 78 years at accelerometer assessment. Condition-specific exclusions were also performed; those with a medical history of a condition were excluded from the analysis specific to that condition (additional information about condition-specific exclusions is shown in eTable 1 in Supplement 1).

### Outcome Definitions

The most common primary causes of noncancer-related hospital admission in the UK Biobank were selected as outcomes for inclusion (eTable 2 in Supplement 1).^16,17^ Some common reasons for hospital admission in this cohort (eg, nausea) were not included because they were not well defined and/or could have reflected diverse underlying conditions. We also included nonmelanoma skin cancer because cancer registry data may have been incomplete for this cancer type.^18^ Although diabetes was not one of the most common primary reasons for hospitalization, this condition was included as an outcome because it is a common secondary reason for admission.^16,17,19^ In total, 25 common conditions were included in the analyses.

Follow-up began after completion of the accelerometer assessment via record linkage to national health records. Incident cases were identified from the primary reason for hospitalization, surgical procedure, or death only, with the exception of diabetes, for which case identification also included any mention on hospital admission records or death certificates. Participants who were admitted to the hospital during follow-up for any of the 25 conditions continued to contribute follow-up time for all other conditions examined until death, end of follow-up, or hospital admission for the condition investigated. Due to the low numbers of deaths recorded for each condition, we referred to cases identified from hospitalization and death records as hospitalizations (eTable 2 in Supplement 1). Further details about health record data, censoring dates, and exclusions are available in the eMethods and eTable 1 in Supplement 1.

### Statistical Analysis

Hazard ratios (HRs) and 95% CIs of hospitalization for each condition per 1-SD increment in mean volume of acceleration were estimated using Cox proportional hazards regression models with stratification by age group and sex. The model used age as the underlying time variable, and we adjusted for self-reported racial and/or ethnic group, body mass index (BMI; calculated as weight in kilograms divided by height in meters squared), socioeconomic status, educational level, employment status, smoking status, alcohol consumption, and several female-specific variables (use of hormone replacement therapy, use of oral contraception, menopausal status, and parity). We controlled for race and ethnicity because these characteristics were potential confounding factors; race and ethnicity categories were White and other (including Asian or Asian British, Black or Black British, multiple races, and other race and/or ethnicity) due to limited representation of participants identifying as other races and ethnicities in this study (Table). We also adjusted for BMI because it may have been a confounder in the associations between physical activity behaviors and disease risk. Adjustment categories were chosen a priori based on previously published analyses,^13,16,17^ and covariate categories are available in the eMethods in Supplement 1. Schoenfeld residuals were used to assess the proportional hazards assumption, and we found no evidence of violation. All tests of significance were 2-sided, and we accounted for multiple testing for the primary analysis using the false discovery rate, which equated to *P* < .03.^20^ We used this same threshold for statistical significance in all subsequent analyses.

**Table.** Participant Characteristics by Fourths of Mean Accelerometer-Measured Physical Activity Levels

Characteristic	Mean accelerometer-measured physical activity by fourths of activity level			
	First (n = 20 430)	Second (n = 20 429)	Third (n = 20 429)	Fourth (n = 20 429)
**Accelerometer-measured physical activity**				
Range of mean accelerometer activity, milligravity	>0.4 to ≤24.0	>24.0 to ≤28.9	>28.9 to ≤34.6	>34.6 to ≤83.8
Mean accelerometer activity, median (IQR), milligravity	21.0 (18.6 to 22.6)	26.5 (25.3 to 27.7)	31.5 (30.1 to 32.9)	39.3 (36.6 to 43.4)
Moderate to vigorous activity, median (IQR), min/d	13.8 (6.0 to 25.0)	22.5 (11.6 to 37.3)	28.9 (15.8 to 46.6)	41.5 (23.5 to 64.7)
**Self-reported physical activity**				
Total MET, median (IQR), min/d	127.7 (53.2 to 266.6)	159.4 (71.6 to 314.6)	187.4 (88.7 to 360.0)	249.3 (124.9 to 456.3)
Moderate to vigorous activity, median (IQR), min/d	17.1 (2.9 to 42.9)	21.4 (5.7 to 51.4)	25.7 (8.6 to 57.1)	34.3 (12.9 to 71.4)
**Anthropometric, sociodemographic, and lifestyle characteristics**				
Sex, No. (%)				
Female	10 291 (50.4)	11 576 (56.7)	12 058 (59.0)	12 128 (59.4)
Male	10 139 (49.6)	8853 (43.3)	8371 (41.0)	8301 (40.6)
Age at accelerometer assessment, mean (SD)	64.0 (7.5)	62.2 (7.7)	60.8 (7.7)	58.9 (7.6)
Race and ethnicity, No. (%)				
White	19 947 (97.6)	19 835 (97.1)	19 806 (97.0)	19 687 (96.4)
Other^a^	483 (2.4)	594 (2.9)	623 (3.0)	742 (3.6)
Height, mean (SD), cm	169.8 (9.3)	169.2 (9.1)	168.8 (9.0)	168.8 (8.9)
BMI, mean (SD)	28.0 (4.8)	26.8 (4.3)	26.1 (4.0)	25.1 (3.7)
Townsend Index score, median (IQR)	−2.4 (−3.8 to 0)	−2.5 (−3.8 to −0.3)	−2.5 (−3.9 to −0.3)	−2.4 (−3.8 to −0.2)
Current smoking, No. (%)	1742 (8.5)	1357 (6.6)	1277 (6.3)	1209 (5.9)
Alcohol consumption at least once/wk, No. (%)	14 482 (70.9)	15 411 (75.4)	15 573 (76.2)	15 626 (76.5)
College degree or equivalent, No. (%)	14 125 (69.1)	14 605 (71.5)	14 644 (71.7)	14 754 (72.2)
Employed or self-employed, No. (%)	10 881 (53.3)	12 644 (61.9)	13 670 (66.9)	14 848 (72.7)

Abbreviations: BMI, body mass index (calculated as weight in kilograms divided by height in meters squared); MET, metabolic equivalent of task.

^a^ Includes Asian or Asian British (n = 891), Black or Black British (n = 676), multiple races (n = 447), and other race and/or ethnicity (n = 428).

To investigate the broader role of physical activity and the subsequent risk of hospitalization, we also estimated the associations between physical activity and the first hospital admission for any condition during the follow-up period. We examined the shape of the dose-response association using restricted cubic splines with knots placed at the 10th, 50th, and 90th percentiles of the distribution, with the reference centered on the mean physical activity level.^21^ In this analysis, mean accelerometer levels higher than the 95th percentile and lower than the 5th percentile were truncated to reduce the consequences of outliers. Models were assessed for nonlinearity using the likelihood ratio test. When models were nonlinear, we displayed the shape of the association using splines; for all other models, linear associations were shown.

To explore the possibility of differing associations by physical activity intensity (sedentary, LPA, and MVPA), we fitted 3 linear models comprising 1-factor, 2-factor, and 3-factor (ie, partition) models per 20-minute-per-day increments.^22^ We also fitted isotemporal models, which included LPA, MVPA, and total wear time to estimate associations when replacing 20 minutes per day of sedentary time with 20 minutes per day of (1) LPA and (2) MVPA, while holding time constant.^22,23^ Twenty minutes per day was used as the time frame because this duration represented an obtainable goal for increasing physical activity and had been used elsewhere.^13^ These models can implicate sleep in some situations (eg, if sedentary activity, LPA, and MVPA are all at high levels for a given individual, then sleep must necessarily be of shorter duration). To ensure short sleep duration was not modifying the associations, we also examined the associations of accelerometer-measured sleep duration with the 25 conditions using 1-factor models.

For each condition, we estimated the proportion of hospitalizations that might have been prevented if participants had increased their MVPA levels by 20 minutes per day. Hospitalization rate using baseline MVPA was estimated using Cox proportional hazards regression models and was calculated as baseline hazard rate multiplied by the relative risk evaluated at the observed MVPA level and covariate levels, summed over each study individual. We then assigned participants a hypothetical additional 20 minutes per day of MVPA; the counterfactual hospitalization rate was estimated using the same method described in the previous sentence. Population-attributable risk was calculated as the baseline hospitalization rate minus the counterfactual rate, divided by the baseline hospitalization rate.^24,25^ Additional information is available in the eMethods in Supplement 1.

For comparability with other published analyses, we repeated the primary analysis by fourths of mean accelerometer-measured physical activity (with the first fourth representing the lowest activity level and the fourth representing the highest; the range of milligravity values for each fourth is shown in the Table). We also estimated HRs for disease risk per 1-SD increment in self-report–based and accelerometer-based mean metabolic equivalent of task.

For the sensitivity analysis, we conducted subgroup analyses for the associations of total accelerometer volume and disease risk stratified by follow-up time (<4 years vs ≥4 years), sex (female vs male), age group at diagnosis (<68 years vs ≥68 years), BMI (<30 vs ≥30), smoking status (never vs ever), and whether the participant’s job involved manual labor (none vs some or more). Subgroups were chosen a priori, and cutoffs for continuous variables were selected based on the median value or clinical classification (ie, BMI). Heterogeneity in the associations for case-specific variables (ie, follow-up time and age at diagnosis) was examined using 2 different subgroups defined by follow-up period, using a χ^2^ test for heterogeneity. Heterogeneity in the associations for the non–case-dependent subgroups was assessed using a χ^2^ interaction term.

To understand the impact of specific adjustments, we sequentially added potential confounders and/or mediators (eg, BMI) to models. We also included further adjustments for diet and health factors.

All analyses were performed using Stata software, version 17.0 (StataCorp LLC), SAS software, version 9.4 (SAS Institute Inc), and R software, version 3.2.3 (R Foundation for Statistical Computing). Figures were created in R software using the Jasper,^26^ survival, and ggplot2 packages.

## Results

Among 81 717 participants, the mean (SD) age at accelerometer assessment was 61.5 (7.9) years; most participants were female (56.4%) and self-identified as White (97.0%). Individuals were followed up over a median (IQR) of 6.8 (6.2-7.3) years, and the median (IQR) time to the first occurrence of each outcome ranged from 2.6 (1.3-4.1) years for female genital prolapse to 4.1 (2.3-5.7) years for ischemic stroke. Ischemic heart disease was the most severe condition (5-year case fatality rate of 1.8%), while many other conditions did not directly cause any deaths (eTable 2 in Supplement 1). Participants with higher total physical activity levels were generally younger and had a lower BMI, and participants with higher proportions were female (Table).

**Figure 1.**
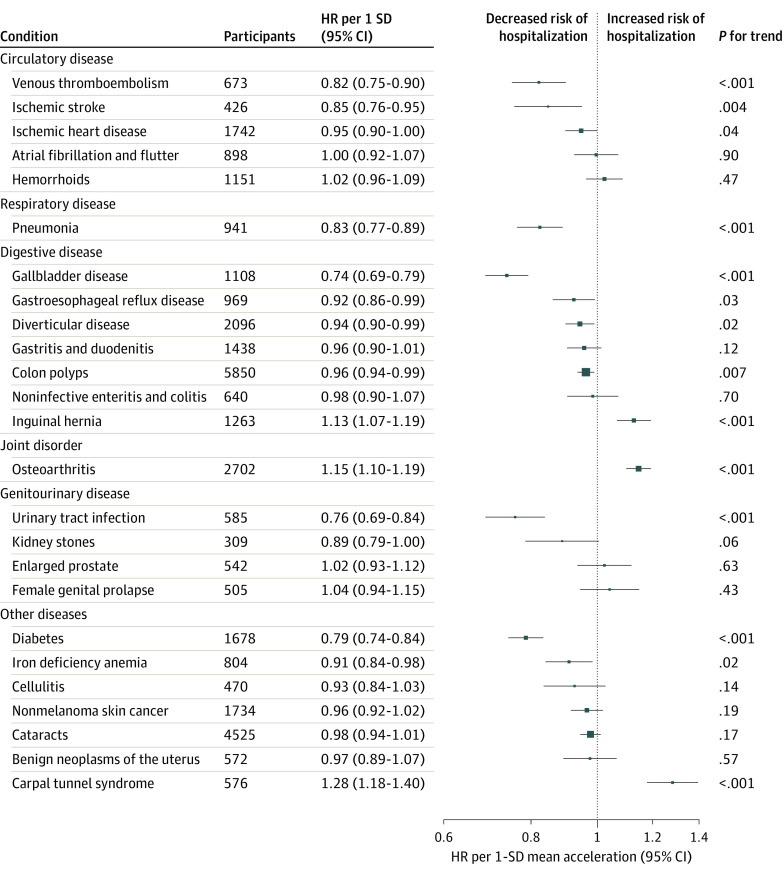
Association of Mean Accelerometer-Measured Physical Activity With Risks of Hospitalization for 25 Common Conditions The SD of accelerometer-measured physical activity was 8.3 milligravity units. Hazard ratios (HRs) and 95% CIs were estimated using Cox proportional hazards regression models with age as the underlying time variable. Models were stratified by age group and sex and adjusted for self-reported racial and/or ethnic group, socioeconomic status, educational level, employment status, smoking status, alcohol consumption, body mass index, and, among female participants, use of oral contraception, menopausal status, and parity. The squares represent HRs, and the horizontal lines represent 95% CIs.

Higher total physical activity was associated with a lower risk of first hospitalization after the accelerometer assessment (48 560 participants admitted to the hospital; HR per 1 SD, 0.96; 95% CI, 0.95-0.97). Higher physical activity was also associated with reduced risks of 9 conditions: gallbladder disease (HR per 1 SD, 0.74; 95% CI, 0.69-0.79), urinary tract infections (UTIs; HR per 1 SD, 0.76; 95% CI, 0.69-0.84), diabetes (HR per 1 SD, 0.79; 95% CI, 0.74-0.84), venous thromboembolism (HR per 1 SD, 0.82; 95% CI, 0.75-0.90), ischemic stroke (HR per 1 SD, 0.85; 95% CI, 0.76-0.95), pneumonia (HR per 1 SD, 0.83; 95% CI, 0.77-0.89), iron deficiency anemia (HR per 1 SD, 0.91; 95% CI, 0.84-0.98), diverticular disease (HR per 1 SD, 0.94; 95% CI, 0.90-0.99), and colon polyps (HR per 1 SD, 0.96; 95% CI, 0.94-0.99). However, higher physical activity was also associated with increased risks of carpal tunnel syndrome (HR per 1 SD, 1.28; 95% CI, 1.18-1.40), osteoarthritis (HR per 1 SD, 1.15; 95% CI, 1.10-1.19), and inguinal hernia (HR per 1 SD, 1.13; 95% CI, 1.07-1.19) (Figure 1). In dose-response analyses, associations were linear, except for pneumonia and UTIs, for which slight positive patterns were observed among individuals with physical activity levels higher than the population mean; however, the risk estimates were imprecise (eFigure 2 in Supplement 1).

**Figure 2.**
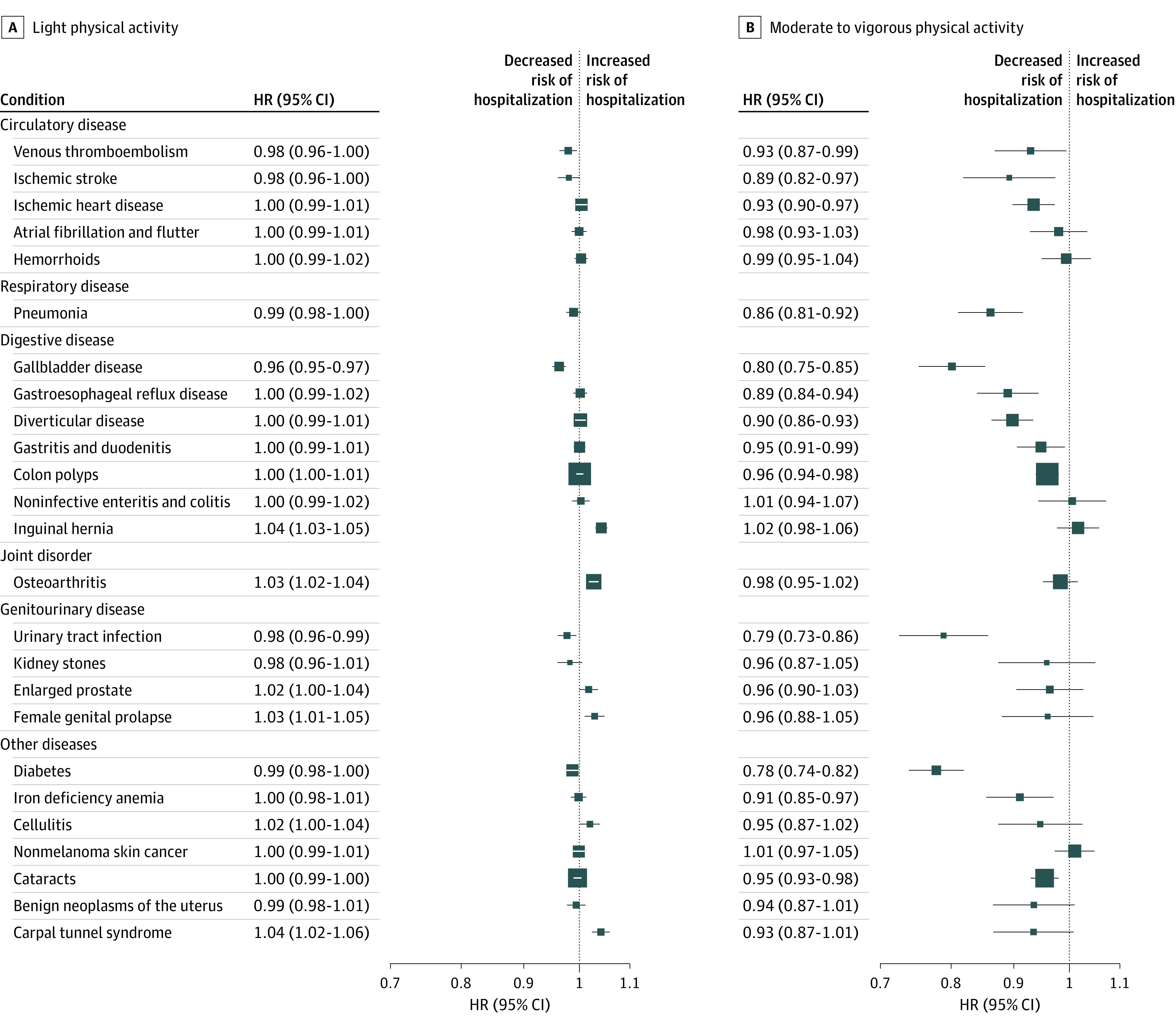
Risks of 25 Conditions When Replacing 20 Minutes per Day of Sedentary Time With 20 Minutes per Day of Light or Moderate to Vigorous Physical Activity Hazard ratios (HRs) and 95% CIs were estimated using Cox proportional hazards regression models with age as the underlying time variable. Models were stratified by age group and sex and adjusted for self-reported racial and/or ethnic group, socioeconomic status, educational level, employment status, smoking status, alcohol consumption, body mass index, and, among female participants, use of hormone replacement therapy, use of oral contraception, menopausal status, and parity, wear time, and time spent doing light and moderate to vigorous intensity physical activity. The squares represent HRs, and the horizontal lines represent 95% CIs.

When inverse associations between mean total physical activity and disease risk were observed, higher sedentary time generally had positive associations with these diseases when using 1-factor models, and higher sedentary time was associated with a lower risk of inguinal hernia (HR per 20-minute-per-day-increment, 0.97; 95% CI, 0.96-0.98), osteoarthritis (HR per 20-minute-per-day increment, 0.99; 95% CI, 0.98-0.99), and female genital prolapse (HR per 20-minute-per-day increment, 0.97; 95% CI, 0.95-0.99) (eTable 3 in Supplement 1). Isotemporal models revealed that substituting 20 minutes per day of sedentary time with 20 minutes per day of MVPA was associated with lower risks of hospitalization for all conditions that were inversely associated with overall physical activity. Substituting 20 minutes per day of LPA yielded less consistent associations (Figure 2). Associations by physical activity intensity were similar in 1-factor, 2-factor, and partition models (eTable 3 in Supplement 1). We observed positive associations between LPA and the risk of osteoarthritis, inguinal hernia, and carpal tunnel syndrome in 1-factor, 2-factor, partition, and isotemporal models (eg, 1-factor model: HR per 20-minute-per-day increment, 1.03 [95% CI, 1.02-1.04] for osteoarthritis, 1.04 [95% CI, 1.03-1.05] for inguinal hernia, and 1.04 [95% CI, 1.02-1.06] for carpal tunnel syndrome). However, for MVPA, the associations appeared null for osteoarthritis and inguinal hernia, and the associations were possibly inverse for carpal tunnel syndrome (eg, 1-factor model: HR per 20 minutes per day of MVPA, 0.93; 95% CI, 0.87-1.01) (Figure 2; eTable 3 in Supplement 1).

Population-attributable risk estimates revealed that increasing MVPA levels by 20 minutes per day was associated with lower hospitalizations, particularly for diabetes (23.0% reduction; 95% CI, 17.1%-28.9%), UTIs (22.7% reduction; 95% CI, 14.8%-30.5%), gallbladder disease (19.8% reduction; 95% CI, 14.0%-25.7%), pneumonia (14.1% reduction; 95% CI, 8.3%-20.0%), iron deficiency anemia (9.4% reduction; 95% CI, 3.5%-15.3%), gastroesophageal reflux disease (9.2% reduction; 95% CI, 3.4%-15.1%), diverticular disease (8.5% reduction; 95% CI, 4.6%-12.4%), cellulitis (8.1% reduction; 95% CI, 0.3%-15.9%), and colon polyps (3.8% reduction; 95% CI, 1.8%-5.7%). Population-attributable risk estimates for the other conditions are shown in eTable 4 in Supplement 1.

Associations between accelerometer-measured physical activity per 1-SD increment in mean metabolic equivalent of task per day and disease risks (eg, diabetes: HR per 1 SD, 0.83; 95% CI, 0.79-0.87) were similar to the mean volume observed in the main analysis (eFigure 3 in Supplement 1). Associations of self-reported physical activity with disease risks were generally directionally consistent but with smaller effect sizes in comparison with accelerometer-measured physical activity (eg, diabetes: HR per 1 SD, 0.96; 95% CI, 0.95-0.97) **(**eFigure 4 in Supplement 1). Associations of mean physical activity by fourths of activity level were consistent with the results found in the main analysis. For example, participants in the highest fourth of physical activity had a 27.1% lower risk of venous thromboembolism (HR per 1 SD, 0.73; 95% CI, 0.57-0.92) compared with those in the lowest fourth of physical activity (eTable 5 in Supplement 1).

### Sensitivity Analysis

The associations of physical activity with hospitalization for each condition were generally consistent across subgroups (significance was defined as *P* < .03 for heterogeneity), although some exceptions were noted (eFigures 5-10 in Supplement 1). Higher physical activity was associated with a reduced risk of cataracts (HR per 1 SD, 0.92; 95% CI, 0.88-0.97) at less than 4 years after accelerometer assessment but not at 4 or more years of follow-up (HR per 1 SD, 1.03; 95% CI, 0.99-1.09; *P* < .001 for heterogeneity) (eFigure 5 in Supplement 1). Physical activity was also associated with a significantly lower risk of pneumonia when duration of follow-up was shorter vs longer (<4 years: HR per 1 SD, 0.76; 95% CI, 0.68-0.84; ≥4 years: HR per 1 SD, 0.92; 95% CI, 0.82-1.03; *P* = .01 for heterogeneity) and when the condition was diagnosed at an older vs younger age (≥68 years: HR per 1 SD, 0.76; 95% CI, 0.69-0.84; <68 years: HR per 1 SD, 0.93; 95% CI, 0.82-1.04; *P* = .01 for heterogeneity) (eFigure 5 and eFigure 6 in Supplement 1). In addition, physical activity was associated with larger HRs for osteoarthritis when duration of follow-up was longer vs shorter (<4 years: HR per 1 SD, 1.08; 95% CI, 1.03-1.14; ≥4 years: HR per 1 SD, 1.25; 95% CI, 1.17-1.34; *P* <.001 for heterogeneity) and when sex was male vs female (men: HR per 1 SD, 1.26; 95% CI, 1.19-1.34; women: HR per 1 SD, 1.06; 95% CI, 1.00-1.12; *P* < .001 for heterogeneity) (eFigure 5 and eFigure 7 in Supplement 1). Physical activity was associated with a lower risk of gastroesophageal reflux disease among women (HR per 1 SD, 0.85; 95% CI, 0.78-0.94) but not men (HR per 1 SD, 1.02; 95% CI, 0.92-1.13; *P* = .01 for heterogeneity) (eFigure 7 in Supplement 1). In addition, physical activity was associated with a significantly higher risk of inguinal hernia among participants without obesity (HR per 1 SD, 1.10; 95% CI, 1.03-1.16), and this risk was greater among those with obesity (HR per 1 SD, 1.39; 95% CI, 1.19-1.62; *P* = .006 for heterogeneity) (eFigure 8 in Supplement 1). Physical activity was associated with significantly lower risks of venous thromboembolism (HR per 1 SD, 0.68; 95% CI, 0.59-0.78) and UTIs (HR per 1 SD, 0.60; 95% CI, 0.52-0.69) among those who had ever smoked but not among those who had never smoked (venous thromboembolism: HR per 1 SD, 0.95; 95% CI, 0.84-1.07; *P* < .001 for heterogeneity; UTIs: HR per 1 SD, 0.95; 95% CI, 0.83-1.08; *P* < .001 for heterogeneity) (eFigure 9 in Supplement 1).

Of the factors we adjusted for, the greatest change in HRs was found when adjusting for BMI, particularly for the risk of diabetes (21.5% decrease), gallbladder disease (12.1% decrease), osteoarthritis (11.7% increase), carpal tunnel syndrome (11.3% increase), and cellulitis (10.7% decrease) (model 5 in eTable 6 in Supplement 1). Risk estimates were not substantially different after further adjustment for medications, hypertension, diet, and self-reported health (models 6-8 in eTable 6 in Supplement 1).

Sleep duration was inversely associated with osteoarthritis (HR per 1 SD, 0.95, 95% CI, 0.91-0.99), inguinal hernia (HR per 1 SD, 0.93; 95% CI, 0.88-0.98), iron deficiency anemia (HR per 1 SD, 0.91; 95% CI, 0.85-0.97), cellulitis (HR per 1 SD, 0.89; 95% CI, 0.81-0.97), and carpal tunnel syndrome (HR per 1 SD, 0.88; 95% CI, 0.81-0.95) and positively associated with gallbladder disease (HR per 1 SD, 1.08; 95% CI, 1.02-1.14) and gastritis and duodenitis (HR per 1 SD, 1.06; 95% CI, 1.01-1.12) (eTable 7 in Supplement 1). These associations would have been generally in the opposite direction if the physical activity associations were explained by the differences in sleep duration between physically active vs sedentary participants.

## Discussion

In this large prospective cohort study, we found that higher levels of accelerometer-measured physical activity were associated with lower risks of hospitalization for 9 of the 25 common conditions (gallbladder disease, UTIs, diabetes, venous thromboembolism, ischemic stroke, pneumonia, iron deficiency anemia, diverticular disease, and colon polyps) examined. While we also observed positive associations between physical activity and inguinal hernia, osteoarthritis, and carpal tunnel syndrome, associations between MVPA and these conditions were null. Substituting 20 minutes per day of sedentary time with 20 minutes per day of MVPA was associated with significant reductions in risk across a broad range of conditions, and we estimated that increasing MVPA time would yield 3.8% to 23.0% lower hospitalizations for some conditions.

The wide range of protective associations observed in these analyses across multiple physiological organ systems highlighted the many benefits of physical activity. Physiological mechanisms that may explain these associations include (1) enhancing immune competency,^27,28^ (2) improving cardiopulmonary health,^29,30,31,32^ (3) improving insulin sensitivity,^33^ (4) controlling cell proliferation,^34^ (5) reducing systemic inflammation,^34,35^ and (6) protecting mitochondrial health.^36^ Higher physical activity levels can also act indirectly by reducing risk factors and health conditions such as body fat and hypertension.

Consistent with previous studies,^2,3,4,37,38,39,40,41^ we found that higher physical activity levels were associated with reduced risks of venous thromboembolism, ischemic stroke, pneumonia, gallbladder disease, colon polyps, diverticular disease, and diabetes. However, we observed generally larger decreases in risk than these other studies.^2,3,4,37,38,39,40,41^ For example, a recent meta-analysis^3^ reported a 13% reduction in the risk of venous thromboembolism for the most active vs least active participants (relative risk, 0.87; 95% CI, 0.79-0.95), whereas we estimated a 27.1% reduction for participants in the highest fourth vs lowest fourth of physical activity, possibly due to the use of self-reported physical activity in the studies included in that meta-analysis. We also observed larger risk reductions for accelerometer-based physical activity than self-reported physical activity. Similarly, we noted differences in the shapes of the dose-response associations. While previous studies have reported curvilinear associations of physical activity with diabetes and ischemic stroke,^37^ we observed linear associations between physical activity and the risks of all conditions except infections (ie, pneumonia and UTIs).

We also observed inverse associations between physical activity and the risks of hospitalization for UTIs and iron deficiency anemia, for which associations are not well characterized in the literature. A previous study^42^ reported that those with higher physical activity levels were less likely to receive a prescription to treat cystitis (the most common type of UTI), but the duration of follow-up was limited (1 year). We are not aware of any study that has investigated the association between physical activity and the risk of clinically diagnosed iron deficiency anemia in the general population.

We found positive associations between LPA and the risks of inguinal hernia, osteoarthritis, and carpal tunnel syndrome. Risks of these conditions have consistently been associated with jobs requiring manual labor, whereas associations with leisure-time activity have been inconsistent.^43,44,45,46,47,48,49^ Future research is needed to categorize the types of LPA that may be underlying factors in these associations and to investigate interventions to manage risks.

### Implications

Our findings reaffirmed the many benefits of physical activity and provided evidence that increasing MVPA levels may help to lower hospital burdens. Our results also highlighted the need for policy makers to implement effective policies to increase physical activity in the population and the important role of clinicians in encouraging patients to increase their physical activity.^50^ Future research should investigate the associations of higher-intensity physical activity with the magnitudes of disease risk and examine the role of different types of physical activity, such as muscle-strengthening exercises.^51^ Identifying high-throughput biomarkers of physical activity will help investigations into the disease-specific mechanisms through which physical activity might be associated with disease risk and aid causal inference.^52^

### Strengths and Limitations

This study has several strengths, with its primary strength being the routine linkage to national hospitalization records, which enables investigations into a broad range of diseases and reduced attrition and misclassification biases. Another strength was the availability of accelerometer data and the distinct calibration of these data to observed behaviors, which improves our ability to categorize different physical activity intensities.^13^ These calibrated data provide robust measurement of total ambulatory physical activity, minimizing the potential for measurement error. By estimating the proportions of hospitalizations that could have been prevented, we provide novel evidence of the possible impact of reducing sedentary time and increasing MVPA levels and deliver a public health message that is more understandable to the general public. Additional strengths include the prospective population-based study design and large sample size. We also restricted incident cases to the primary reason for hospitalization or death only (except for diabetes); thus, we were more likely to identify more clinically meaningful cases, which present the greatest burden to health care services.

This study also has limitations. Our study is observational; therefore, although we controlled for many relevant measured confounders, we cannot fully exclude the possibility of residual confounding or reverse causation. Poor health and mobility may be a barrier to physical activity, while some diagnoses may lead participants to increase their activity for disease control.^53,54^ Both low physical activity and poor health are associated with the risk of hospitalization; therefore, these complex associations are difficult to fully distentangle.^55^ Although wrist-worn accelerometers have a high level of precision and the risk of biases from nonwear time and technical error is low, these accelerometers do not fully capture nonambulatory activities, such as indoor cycling or weightlifting.^11,12^ In addition, it is unclear to what extent 1-week measurements are representative of habitual physical activity over longer periods. Studies have reported moderate to high correlations (eg, *r* = 0.54-0.82) for repeat assessments of wearable devices over durations ranging from 6 months to 4 years.^56,57,58^ If this measurement does not represent habitual activity levels, underestimation of risk estimates could occur.^59^ Participant characteristics were collected for a mean of 5.7 years before the accelerometer measurement, although repeat measurements of these covariates in subsets of this cohort have been found to be stable over time.^60^ The UK Biobank participants are predominantly of White European ancestry and are healthier than the sampling population; therefore, the magnitude of risk estimates may not be generalizable to some other populations.^61,62,63^

## Conclusions

This large prospective cohort study found protective associations between physical activity and the risks of cardiopulmonary diseases, gallbladder disease, and diabetes, and novel associations with other conditions were observed across a wide range of physiological systems. These results suggest that increasing MVPA levels by 20 minutes per day could yield substantial reductions in hospitalizations and may be a useful nonpharmaceutical intervention to reduce health care burdens and improve quality of life.

## Notes

Supplement 1. **eMethods.** Self-Reported Physical Activity, Outcome Definitions, Covariate Adjustment Categories, and Population Attributable Risk **eFigure 1.** Participant Exclusion Criteria **eFigure 2.** Hazard Ratios and 95% CIs for Associations Between Accelerometer-Measured Physical Activity and Risks of 25 Common Conditions in the UK Biobank **eFigure 3.** Associations of Accelerometer-Measured Physical Activity per 1 SD Increment in Mean METs With Risks of 25 Common Conditions **eFigure 4.** Associations per 1 SD Increment in Self-Reported Physical Activity (MET Hours/Week) With Risks of 25 Common Conditions **eFigure 5.** Associations of Mean Accelerometer-Measured Physical Activity (Milligravity Units) With Risks of 25 Common Conditions Stratified by Length of Follow-up **eFigure 6.** Associations of Mean Accelerometer-Measured Physical Activity (Milligravity Units) With Risks of 25 Common Conditions Stratified by Age at Diagnosis **eFigure 7.** Associations of Accelerometer-Measured Physical Activity (Milligravity Units) With Risks of 25 Common Conditions Stratified by Sex **eFigure 8.** Associations of Accelerometer-Measured Physical Activity (Milligravity Units) With Risks of 25 Common Conditions Stratified by Obesity Status **eFigure 9.** Associations of Accelerometer-Measured Physical Activity (Milligravity Units) With Risks of 25 Common Conditions Stratified by Smoking Status **eFigure 10.** Associations of Accelerometer-Measured Physical Activity (Milligravity Units) With Risks of 25 Common Conditions Stratified by Whether Job Requires Manual Labor **eTable 1.** Disease Outcome Definitions and Exclusion Criteria **eTable 2.** Disease Definitions and 5-Year Case Fatality Rate **eTable 3.** Associations of Time Spent Doing Sedentary, Light, and Moderate to Vigorous Physical Activity With Risks of 25 Common Conditions **eTable 4.** Estimated Percentage of Hospitalizations Potentially Prevented by Increasing Moderate to Vigorous Physical Activity by 20 Minutes per Day **eTable 5.** Associations of Mean Accelerometer-Measured Physical Activity (Fourths) With Risks of 25 Common Conditions **eTable 6.** Sequential Model Adjustments for Mean Accelerometer-Measured Physical Activity (Milligravity Units) and Risks of 25 Common Conditions **eTable 7.** Associations of Accelerometer-Measured Time Spent Sleeping With Risks of 25 Common Conditions
eReferences

Supplement 2.
Data Sharing Statement
