# Supplementary material for: Association of Accelerometer-Measured Physical Activity Level With Risks of Hospitalization for 25 Common Health Conditions in UK Adults
Source: JAMA Netw Open. 2023 Feb 16;6(2):e2256186. doi: 10.1001/jamanetworkopen.2022.56186 (PMC9936337; doi:10.1001/jamanetworkopen.2022.56186)
Supplement: Supplement 1. — eMethods. Self-Reported Physical Activity, Outcome Definitions, Covariate Adjustment Categories, and Population Attributable Risk eFigure 1. Participant Exclusion Criteria eFigure 2. Hazard Ratios and 95% CIs for Associations Between Accelerometer-Measured Physical Activity and Risks of 25 Common Conditions in the UK Biobank eFigure 3. Associations of Accelerometer-Measured Physical Activity per 1 SD Increment in Mean METs With Risks of 25 Common Conditions eFigure 4. Associations per 1 SD Increment in Self-Reported Physical Activity (MET Hours/Week) With Risks of 25 Common Conditions eFigure 5. Associations of Mean Accelerometer-Measured Physical Activity (Milligravity Units) With Risks of 25 Common Conditions Stratified by Length of Follow-up eFigure 6. Associations of Mean Accelerometer-Measured Physical Activity (Milligravity Units) With Risks of 25 Common Conditions Stratified by Age at Diagnosis eFigure 7. Associations of Accelerometer-Measured Physical Activity (Milligravity Units) With Risks of 25 Common Conditions Stratified by Sex eFigure 8. Associations of Accelerometer-Measured Physical Activity (Milligravity Units) With Risks of 25 Common Conditions Stratified by Obesity Status eFigure 9. Associations of Accelerometer-Measured Physical Activity (Milligravity Units) With Risks of 25 Common Conditions Stratified by Smoking Status eFigure 10. Associations of Accelerometer-Measured Physical Activity (Milligravity Units) With Risks of 25 Common Conditions Stratified by Whether Job Requires Manual Labor eTable 1. Disease Outcome Definitions and Exclusion Criteria eTable 2. Disease Definitions and 5-Year Case Fatality Rate eTable 3. Associations of Time Spent Doing Sedentary, Light, and Moderate to Vigorous Physical Activity With Risks of 25 Common Conditions eTable 4. Estimated Percentage of Hospitalizations Potentially Prevented by Increasing Moderate to Vigorous Physical Activity by 20 Minutes per Day eTable 5. Associations of Mean Accelerometer-Measured Ph [file jamanetwopen-e2256186-s001.pdf]

Supplementary Online Content

Watts EL, Saint-Maurice PF, Doherty A, et al. Association of accelerometer-measured physical activity level with risks of hospitalization for 25 common health conditions in UK adults. *JAMA Netw Open*. 2023;6(2):e2256186. doi:10.1001/jamanetworkopen.2022.56186

eMethods. Self-Reported Physical Activity, Outcome Definitions, Covariate Adjustment Categories, and Population Attributable Risk

eFigure 1. Participant Exclusion Criteria

eFigure 2. Hazard Ratios and 95% CIs for Associations Between Accelerometer-Measured Physical Activity and Risks of 25 Common Conditions in the UK Biobank

eFigure 3. Associations of Accelerometer-Measured Physical Activity per 1 SD Increment in Mean METs With Risks of 25 Common Conditions

eFigure 4. Associations per 1 SD Increment in Self-Reported Physical Activity (MET Hours/Week) With Risks of 25 Common Conditions

eFigure 5. Associations of Mean Accelerometer-Measured Physical Activity (Milligravity Units) With Risks of 25 Common Conditions Stratified by Length of Follow-up

eFigure 6. Associations of Mean Accelerometer-Measured Physical Activity (Milligravity Units) With Risks of 25 Common Conditions Stratified by Age at Diagnosis

eFigure 7. Associations of Accelerometer-Measured Physical Activity (Milligravity Units) With Risks of 25 Common Conditions Stratified by Sex

eFigure 8. Associations of Accelerometer-Measured Physical Activity (Milligravity Units) With Risks of 25 Common Conditions Stratified by Obesity Status

eFigure 9. Associations of Accelerometer-Measured Physical Activity (Milligravity Units) With Risks of 25 Common Conditions Stratified by Smoking Status

eFigure 10. Associations of Accelerometer-Measured Physical Activity (Milligravity Units) With Risks of 25 Common Conditions Stratified by Whether Job Requires Manual Labor

eTable 1. Disease Outcome Definitions and Exclusion Criteria

eTable 2. Disease Definitions and 5-Year Case Fatality Rate

eTable 3. Associations of Time Spent Doing Sedentary, Light, and Moderate to Vigorous Physical Activity With Risks of 25 Common Conditions

eTable 4. Estimated Percentage of Hospitalizations Potentially Prevented by Increasing Moderate to Vigorous Physical Activity by 20 Minutes per Day

eTable 5. Associations of Mean Accelerometer-Measured Physical Activity (Fourths) With Risks of 25 Common Conditions

eTable 6. Sequential Model Adjustments for Mean Accelerometer-Measured Physical Activity (Milligravity Units) and Risks of 25 Common Conditions

eTable 7. Associations of Accelerometer-Measured Time Spent Sleeping With Risks of 25 Common Conditions

eReferences

This supplementary material has been provided by the authors to give readers additional information about their work.

© 2023 Watts EL et al. *JAMA Network Open*.

eMethods. Self-Reported Physical Activity, Outcome Definitions, Covariate Adjustment Categories, and Population Attributable Risk

Self-reported physical activity

Self-reported physical activity was assessed using questions adapted from the International Physical Activity Questionnaire (IPAQ) short form, a validated survey based on the frequency and duration of walking, moderate and vigorous activity[1]. To estimate total metabolic equivalent of tasks (MET) hours per week of physical activity (energy expenditure relative to quiet sitting), the durations of each level of activity was weighted by estimated MET values (3.3, 4.0 and 8.0 METs for walking, moderate, and vigorous intensity, respectively). Following IPAQ guidelines, physical activity for any category of less than 10 min per day was recoded to 0 and durations of >180 min per day were truncated[1].

Outcome definitions

Hospital admission data includes any patient who is admitted to the hospital and occupies a bed (both for emergency and planned admissions) but does not include outpatients or accident and emergency (unless patient is subsequently admitted), and we did not include maternity and administrative psychiatry fields (stored in separate datasets by UK Biobank). More information for these data is available from: https://biobank.ndph.ox.ac.uk/showcase/showcase/docs/HospitalEpisodeStatistics.pdf and published elsewhere[2].

For participants in England, Hospital Episode Statistics (HES) and information on date and cause of death were available until 30th September 2021. For participants in Scotland, the Scottish Morbidity Records and information on date and cause of death were available until 31st July 2020. For participants in Wales, the Patient Episode Database and information on date and cause of death were available until 28th February 2018. Cancer registry data were also available from the NHS Central Registers.

Disease endpoints, information on diagnoses or procedures associated with hospital admissions, and causes of death were all coded according to the 9th or 10th revisions of the World Health Organization's International

© 2023 Watts EL et al. *JAMA Network Open*.

Classification of Diseases (ICD-9 and ICD-10), and the Office of Population Censuses and Surveys classification of surgical operations and procedures (OPCS-4), fourth revision.

The most common primary causes of non-cancer related hospital admission in the UK Biobank population were selected as outcomes for inclusion in this analysis. Some common reasons for hospital admission in this cohort (e.g., nausea) were not included because they were not well-defined and/or could reflect diverse underlying conditions. The top 25 reasons for hospital admission in the UK Biobank population were generally similar to the English records although some differences existed, for instance English national hospital records have higher numbers of dental caries and tonsilitis admissions, likely relating to the age of the cohort participants[3]. UK Biobank maintains tabulations of summary hospital diagnoses, which are available from the data showcase (https://biobank.ndph.ox.ac.uk/showcase/field.cgi?id=41270).

For each condition, we excluded participants with a relevant diagnosis or procedure prior to recruitment, ascertained through the touchscreen questionnaire, nurse-guided interviews, and hospital admission data (ever recorded). For benign growths of the uterus and colon, cases diagnosed within one year of a concurrent uterine cancer or colorectal cancer, respectively, were excluded to remove the possibility of misclassification. Participants with a hospital record of ischemic heart disease, atrial fibrillation and flutter, ischemic stroke or venous thromboembolism were excluded from any of the cardiovascular disease analyses, as these conditions are risk factors for each other, and diagnosis might affect physical activity levels. Those who were admitted to hospital for any condition during follow-up were still eligible to become a case for any condition. Disease codes and exclusion criteria are available from **eTable 1**.

To provide an indicator of relative severity, we calculated the 5-year case fatality rate for each condition (**eTable 2)**.

© 2023 Watts EL et al. *JAMA Network Open*.

Covariate adjustment categories

HRs and 95% CIs were estimated using Cox regression with age as the underlying time variable. Models were stratified by age group (< 50, 50–54, 55–59, 60–64, 65-70, and ≥ 70 years), and sex and adjusted for self-reported racial/ethnic group (White, other), socioeconomic status (Townsend index, fifths), education level (College or university degree/vocational qualification, further education, school leaver), employment (paid/self-employment, not employment, retired), smoking status (never, previous, current moderate (<15 cigarettes /day), current heavy (≥15 cigarettes /day), current unknown), alcohol consumption frequency (never, <3, 3+ /wk), BMI (<25, 25.0-29.9, 30.0-34.9, 35+ kg/m^2), and for females: HRT use (current, former, never), oral contraceptive pill (current, former, never), menopause status (yes, no), and parity (none, 1-2, 3+).

Population attributable risk

MVPA was categorized as (<20 minutes/day, 20-40, 40-60, 60-80, 80-100, 100-120, 120-140, 140 +). Age was the underlying time variable and models were adjusted for age, sex, self-reported racial/ethnic group (White, other), socioeconomic status (Townsend index, fifths), education level (College or university degree/vocational qualification, further education, school leaver), employment (paid/self-employment, not employment, retired), smoking status (never, previous, current moderate (<15 cigarettes /day), current heavy (≥15 cigarettes /day), current unknown), alcohol consumption frequency (never, <3, 3+ /wk), BMI (<25, 25.0-29.9, 30.0-34.9, 35+ kg/m^2), and for females: HRT use (current, former, never), oral contraceptive pill (current, former, never), menopause status (yes, no), parity (none, 1-2, 3+).

Rates of hospitalization by baseline accelerometer measured MVPA was estimated using Cox regression models $R = I\sum r_i$, where I was the baseline hazard rate and r_i were the relative risk evaluated at the measured activity level and covariate levels of each study individual i, and the sum is over all the study individuals.

© 2023 Watts EL et al. *JAMA Network Open*.

We then assigned participants an additional 20-min/day of MVPA, using the same categories as described above, such that some participants will move up into the next category of MVPA. Hospitalization rate using the counterfactual physical activity level was computed using the formula: $R^*=I\sum r_i^*$, where r_i^* represents the counterfactual disease risk. Population attributable risk (PAR) was calculated as (R-R*)/R[4, 5], and the baseline hazard rate I cancels out of this expression.

© 2023 Watts EL et al. *JAMA Network Open*.

eFigure 1. Participant Exclusion Criteria

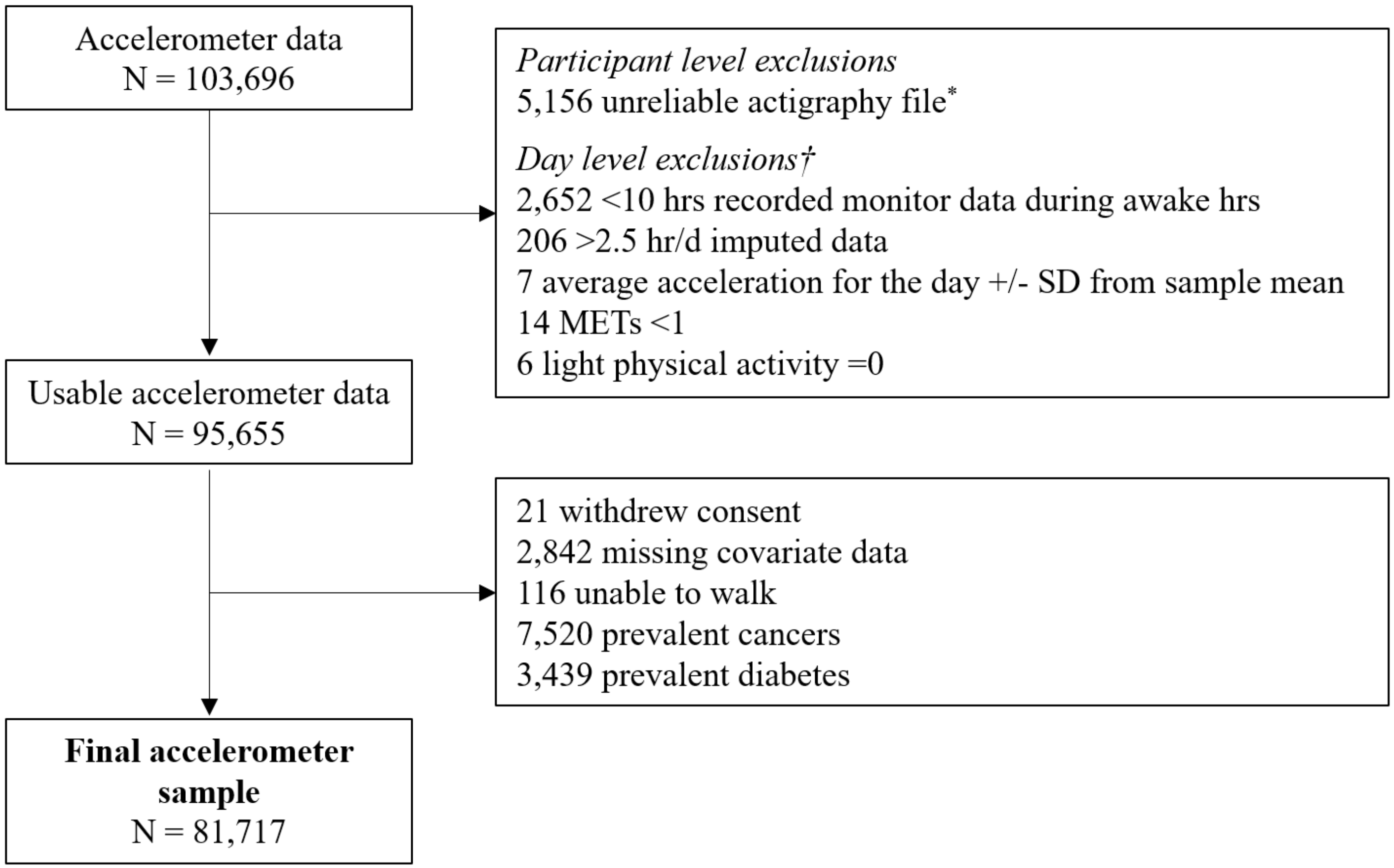

*Includes datasets lost, files that were damaged or of low size (Field 90002), not calibrated (Field 90016), or with unexpected large number of readings exceeding =/-8g sensor (Fields 90183 and 90185)

†Number of participants excluded after day-level criteria applied

© 2023 Watts EL et al. *JAMA Network Open*.

eFigure 2. Hazard Ratios and 95% CIs for Associations Between Accelerometer-Measured Physical Activity and Risks of 25 Common Conditions in the UK Biobank

Circulatory diseases

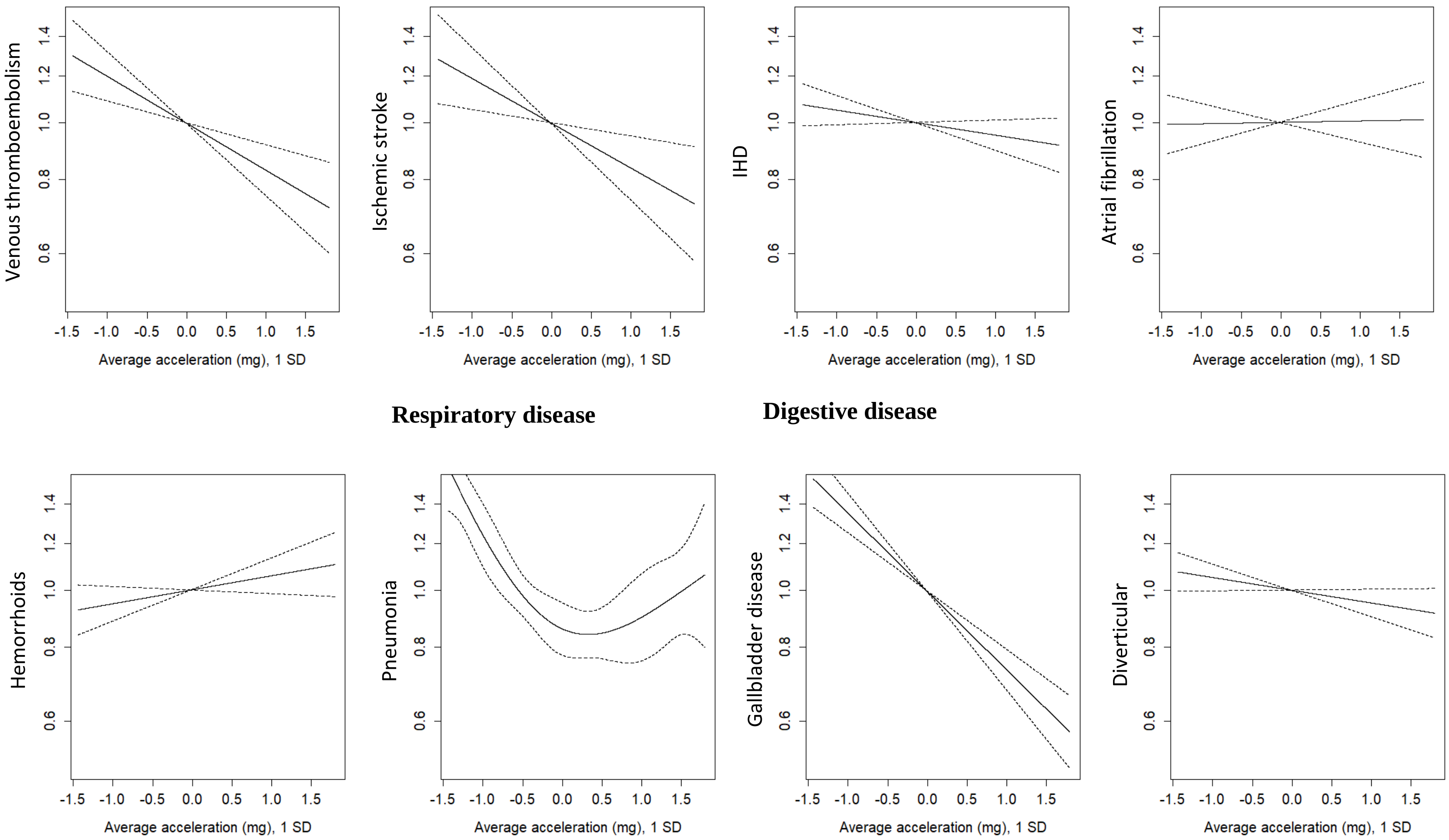

Cubic splines used when they demonstrated significant improvement to linear model fit (tested using the likelihood ratio test), else linear relationships are displayed. Physical activity levels higher and lower than the 95th and 5th percentile, respectively, were truncated. HRs and 95% CIs were estimated using Cox regression with age as the underlying time variable. Models were stratified by age group (< 50, 50–54, 55–59, 60–64, 65-70, and ≥ 70 years), and sex and adjusted for self-reported racial/ethnic group (non-White, White), socioeconomic status (Townsend index, fifths), education level (College or university degree/vocational qualification, further education, school leaver), employment (paid/self-employment, not employment, retired), smoking status (never, previous, current (<15 cigarettes per day), current (≥15 cigarettes per day), current unknown), alcohol consumption frequency (never, <3, 3+ /wk), BMI (<25, 25.0-29.9, 30.0-34.9, 35+ kg/m^2), and for females: HRT use (current, former, never), oral contraceptive pill (current, former, never), menopause status (yes, no), parity (none, 1-2, 3+). Solid line represents the hazard ratio and the dotted lines, the 95% confidence intervals.

Abbreviations: IHD = ischemic heart disease; HRT= hormone replacement therapy; SD = standard deviation.

© 2023 Watts EL et al. *JAMA Network Open*.

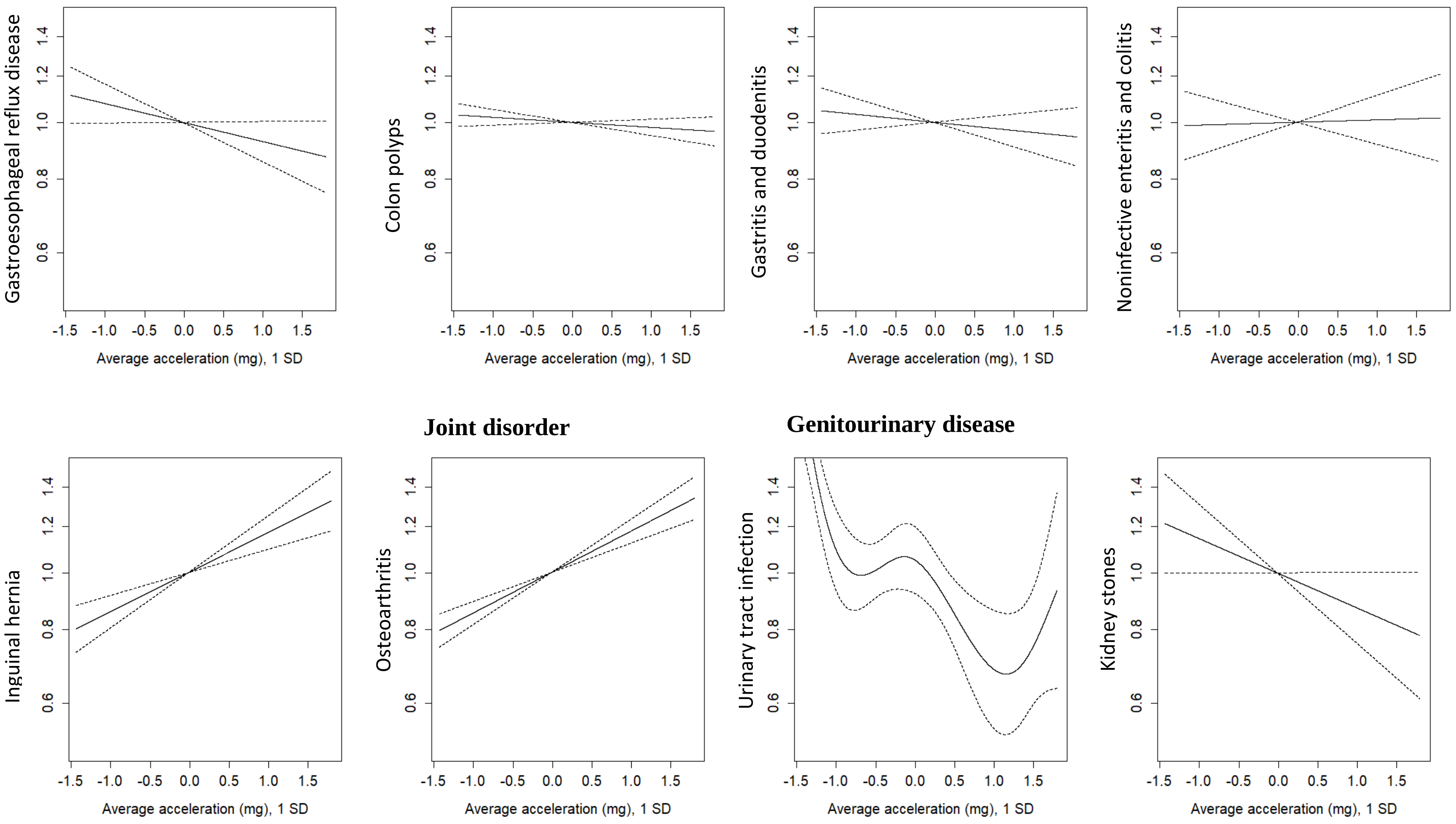

eFigure 2 (continued): Hazard ratios and 95% confidence intervals for associations between accelerometer-measured physical activity and risks of 25 common conditions in the UK Biobank.
Cubic splines used when they demonstrated significant improvement to linear model fit (tested using the likelihood ratio test), else linear relationships are displayed. Physical activity levels higher and lower than the 95th and 5th percentile, respectively, were truncated. HRs and 95% CIs were estimated using Cox regression with age as the underlying time variable. Models were stratified by age group (< 50, 50–54, 55–59, 60–64, 65-70, and ≥ 70 years), and sex and adjusted for self-reported racial/ethnic group (non-White, White), socioeconomic status (Townsend index, fifths), education level (College or university degree/vocational qualification, further education, school leaver), employment (paid/self-employment, not employment, retired), smoking status (never, previous, current moderate (<15 cigarettes per day), current heavy (≥15 cigarettes per day), current unknown), alcohol consumption frequency (never, <3, 3+ /wk), BMI (<25, 25.0-29.9, 30.0-34.9, 35+ kg/m^2), and for females: HRT use (current, former, never), oral contraceptive pill (current, former, never), menopause status (yes, no), parity (none, 1-2, 3+). Solid line represents the hazard ratio and the dotted lines, the 95% confidence intervals.
Abbreviations: HRT= hormone replacement therapy; IHD = ischemic heart disease; SD = standard deviation.

© 2023 Watts EL et al. *JAMA Network Open*.

Other disease

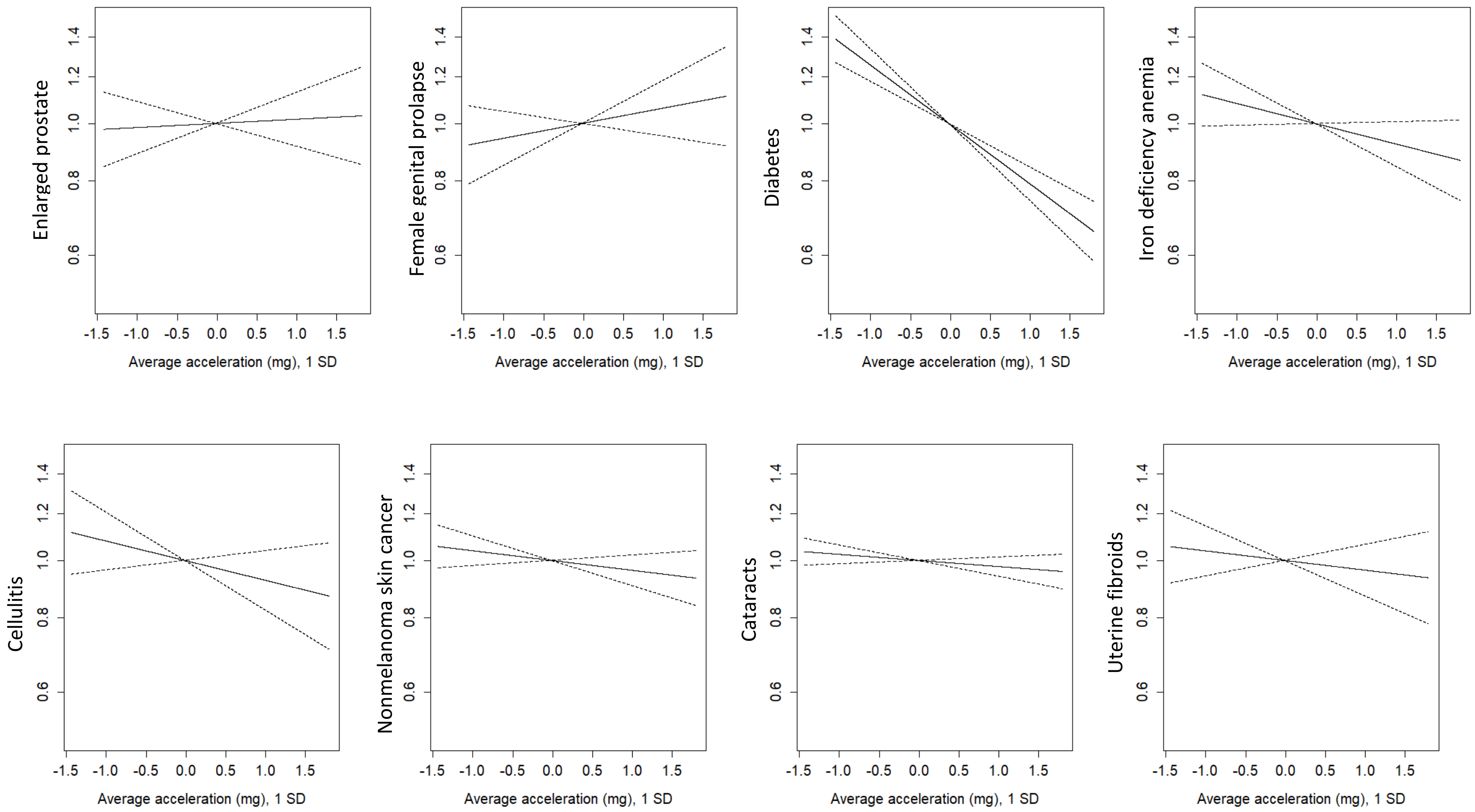

eFigure 2 (continued): Hazard ratios and 95% confidence intervals for associations between accelerometer-measured physical activity and risks of 25 common conditions in the UK Biobank.
Cubic splines used when they demonstrated significant improvement to linear model fit (tested using the likelihood ratio test), else linear relationships are displayed. Physical activity levels higher and lower than the 95th and 5th percentile, respectively, were truncated. HRs and 95% CIs were estimated using Cox regression with age as the underlying time variable. Models were stratified by age group (< 50, 50–54, 55–59, 60–64, 65-70, and ≥ 70 years), and sex and adjusted for self-reported racial/ethnic group (non-White, White), socioeconomic status (Townsend index, fifths), education level (College or university degree/vocational qualification, further education, school leaver), employment (paid/self-employment, not employment, retired), smoking status (never, previous, current (<15 cigarettes per day), current (≥15 cigarettes per day), current unknown), alcohol consumption frequency (never, <3, 3+ /wk), BMI (<25, 25.0-29.9, 30.0-34.9, 35+ kg/m^2), and for females: HRT use (current, former, never), oral contraceptive pill (current, former, never), menopause status (yes, no), parity (none, 1-2, 3+). Solid line represents the hazard ratio and the dotted lines, the 95% confidence intervals.
Abbreviations: HRT= hormone replacement therapy; IHD = ischemic heart disease; SD = standard deviation.

© 2023 Watts EL et al. *JAMA Network Open*.

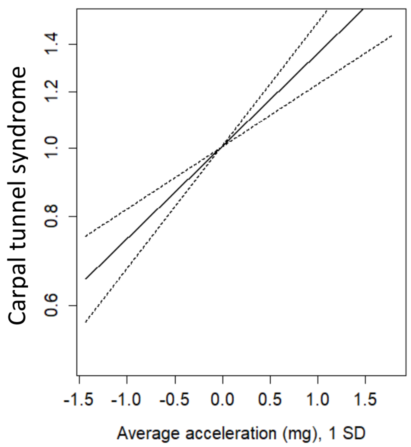

eFigure 2 (continued): Hazard ratios and 95% confidence intervals for associations between accelerometer-measured physical activity and risks of 25 common conditions in the UK Biobank.
Cubic splines used when they demonstrated significant improvement to linear model fit (tested using the likelihood ratio test), else linear relationships are displayed. Physical activity levels higher and lower than the 95th and 5th percentile, respectively, were truncated. HRs and 95% CIs were estimated using Cox regression with age as the underlying time variable. Models were stratified by age group (< 50, 50–54, 55–59, 60–64, 65-70, and ≥ 70 years), and sex and adjusted for self-reported racial/ethnic group (non-White, White), socioeconomic status (Townsend index, fifths), education level (College or university degree/vocational qualification, further education, school leaver), employment (paid/self-employment, not employment, retired), smoking status (never, previous, current moderate (<15 cigarettes per day), current heavy (≥15 cigarettes per day), current unknown), alcohol consumption frequency (never, <3, 3+ /wk), BMI (<25, 25.0-29.9, 30.0-34.9, 35+ kg/m^2), and for females: HRT use (current, former, never), oral contraceptive pill (current, former, never), menopause status (yes, no), parity (none, 1-2, 3+). Solid line represents the hazard ratio and the dotted lines, the 95% confidence intervals.
Abbreviations: HRT= hormone replacement therapy; IHD = ischemic heart disease; SD = standard deviation.

© 2023 Watts EL et al. *JAMA Network Open*.

eFigure 3. Associations of Accelerometer-Measured Physical Activity per 1 SD Increment in Mean METs With Risks of 25 Common Conditions*

Condition	Cases	HR per 1 SD (95% CI)	Ptrend
**Circulatory disease**			
Venous thromboembolism	673	0.88 (0.82, 0.96)	**0.002**
Ischemic stroke	426	0.85 (0.77, 0.94)	**0.002**
Ischemic heart disease	1742	0.96 (0.92, 1.01)	0.10
Atrial fibrilliation and flutter	898	1.01 (0.94, 1.08)	0.84
Hemorrhoids	1151	1.00 (0.95, 1.06)	0.90
**Respiratory disease**			
Pneumonia	941	0.90 (0.84, 0.96)	**0.001**
**Digestive disease**			
Gallbladder disease	1108	0.76 (0.72, 0.81)	**<0.0001**
Gastro-esophageal reflux disease	969	0.91 (0.85, 0.97)	**0.003**
Diverticular disease	2096	0.92 (0.88, 0.96)	**0.0003**
Gastritis and duodentis	1438	0.94 (0.89, 0.99)	**0.02**
Colon polyps	5850	0.97 (0.95, 1.00)	**0.02**
Non-infective enteritis and colitis	640	1.00 (0.92, 1.08)	0.93
Inguinal hernia	1263	1.14 (1.09, 1.20)	**<0.0001**
**Joint disorder**			
Osteoarthritis	2702	1.08 (1.04, 1.12)	**0.0001**
**Genitourinary disease**			
Urinary tract infection	585	0.80 (0.73, 0.87)	**<0.0001**
Kidney stones	309	0.91 (0.81, 1.01)	0.08
Enlarged prostate	542	1.03 (0.95, 1.11)	0.48
Female genital prolapse	505	1.04 (0.95, 1.15)	0.36
**Other diseases**			
Diabetes	1678	0.83 (0.79, 0.87)	**<0.0001**
Iron deficiency anemia	804	0.97 (0.90, 1.04)	0.37
Cellulitis	470	1.07 (0.98, 1.17)	0.16
Non melanoma skin cancer	1734	0.99 (0.94, 1.04)	0.63
Cataracts	4525	0.96 (0.93, 0.99)	**0.01**
Benign neoplasms of the uterus	572	0.95 (0.87, 1.03)	0.23
Carpal tunnel syndrome	576	1.13 (1.04, 1.22)	**0.004**

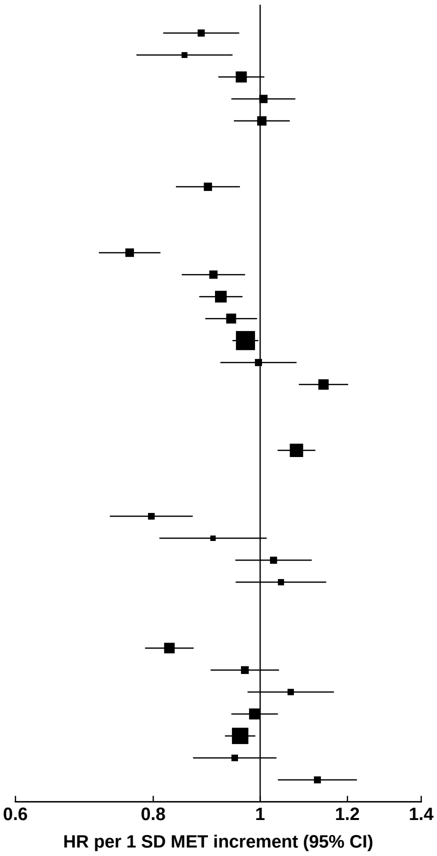

HRs and 95% CIs were estimated using Cox regression with age as the underlying time variable. Models were stratified by age group (< 50, 50–54, 55–59, 60–64, 65-70, and ≥ 70 years), and sex and adjusted for self-reported racial/ethnic group (non-White, White), socioeconomic status (Townsend index, fifths), education level (College or university degree/vocational qualification, further education, school leaver), employment (paid/self-employment, not employment, retired), smoking status (never, previous, current moderate (<15 cigarettes per day), current heavy (≥15 cigarettes per day), current unknown), alcohol consumption frequency (never, <3, 3+ /wk), BMI (<25, 25.0-29.9, 30.0-34.9, 35+ kg/m^2), and for females: HRT use (current, former, never), oral contraceptive pill (current, former, never), menopause status (yes, no), parity (none, 1-2, 3+). The boxes represent the HRs, and vertical lines represent 95% CIs, p-values are bold where p<0.03.

*1 MET SD = 0.09.

Abbreviations: CI=confidence interval; HR=hazard ratio; HRT= hormone replacement therapy; MET=metabolic equivalent of task; SD=standard deviation

© 2023 Watts EL et al. *JAMA Network Open*.

eFigure 4. Associations per 1 SD Increment in Self-Reported Physical Activity (MET Hours/Week) With Risks of 25 Common Conditions*

Condition	Cases	HR per 1 SD (95% CI)	Ptrend
**Circulatory disease**			
Venous thromboembolism	6121	1.01 (0.99, 1.03)	0.32
Ischemic stroke	4084	1.02 (1.00, 1.05)	0.10
Ischemic heart disease	17606	1.01 (0.99, 1.02)	0.38
Atrial fibrilliation and flutter	7457	1.02 (1.00, 1.04)	0.06
Hemorrhoids	11870	1.00 (0.98, 1.01)	0.86
**Respiratory disease**			
Pneumonia	9897	0.96 (0.95, 0.98)	**0.0001**
**Digestive disease**			
Gallbladder disease	11613	0.95 (0.93, 0.96)	**<0.0001**
Gastro-esophageal reflux disease	9319	1.01 (0.99, 1.02)	0.51
Diverticular disease	18814	1.00 (0.99, 1.01)	0.89
Gastritis and duodentis	14586	0.99 (0.98, 1.01)	0.41
Colon polyps	50160	0.98 (0.97, 0.99)	**<0.0001**
Non-infective enteritis and colitis	7441	0.98 (0.96, 1.00)	0.05
Inguinal hernia	11927	1.05 (1.04, 1.07)	**<0.0001**
**Joint disorder**			
Osteoarthritis	25322	1.11 (1.09, 1.12)	**<0.0001**
**Genitourinary disease**			
Urinary tract infection	7085	0.95 (0.93, 0.97)	**<0.0001**
Kidney stones	3084	1.00 (0.97, 1.03)	0.98
Enlarged prostate	4923	0.97 (0.95, 1.00)	**0.02**
Female genital prolapse	6674	1.03 (1.00, 1.05)	**0.02**
**Other diseases**			
Diabetes	16643	0.96 (0.95, 0.97)	**<0.0001**
Iron deficiency anemia	6674	0.97 (0.95, 1.00)	**0.03**
Cellulitis	4719	1.03 (1.00, 1.05)	0.05
Non melanoma skin cancer	13541	1.00 (0.99, 1.02)	0.86
Cataracts	33458	0.97 (0.97, 0.98)	**<0.0001**
Benign neoplasms of the uterus	6767	1.00 (0.98, 1.03)	0.99
Carpal tunnel syndrome	5963	1.12 (1.10, 1.14)	**<0.0001**

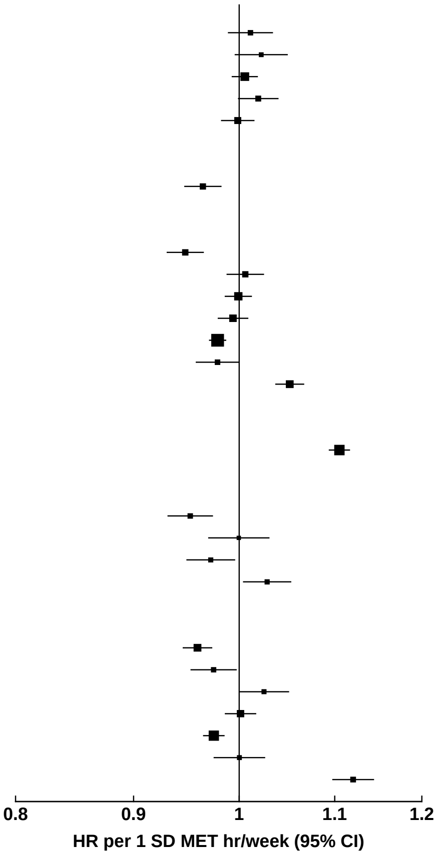

*SD=32.2

HRs and 95% CIs were estimated using Cox regression with age as the underlying time variable. Models were stratified by age group (< 45, 45–49, 50–54, 55–59, 60-64, and ≥ 65 years), and sex and adjusted for self-reported racial/ethnic group (White, other), socioeconomic status (Townsend index, fifths), education level (College or university degree/vocational qualification, further education, school leaver), employment (paid/self-employment, not employment, retired), smoking status (never, previous, current moderate (<15 cigarettes per day), current heavy (≥15 cigarettes per day), current unknown), alcohol consumption frequency (never, <3, 3+ /wk), BMI (<25, 25.0-29.9, 30.0-34.9, 35+ kg/m^2), and for females: HRT use (current, former, never), oral contraceptive pill (current, former, never), menopause status (yes, no), parity (none, 1-2, 3+). The boxes represent the HRs, and vertical lines represent 95% CIs, p-values are bold where p<0.03.

Abbreviations: CI=confidence interval; HR=hazard ratio; HRT= hormone replacement therapy

© 2023 Watts EL et al. *JAMA Network Open*.

eFigure 5. Associations of Mean Accelerometer-Measured Physical Activity (Milligravity Units) With Risks of 25 Common Conditions Stratified by Length of Follow-up

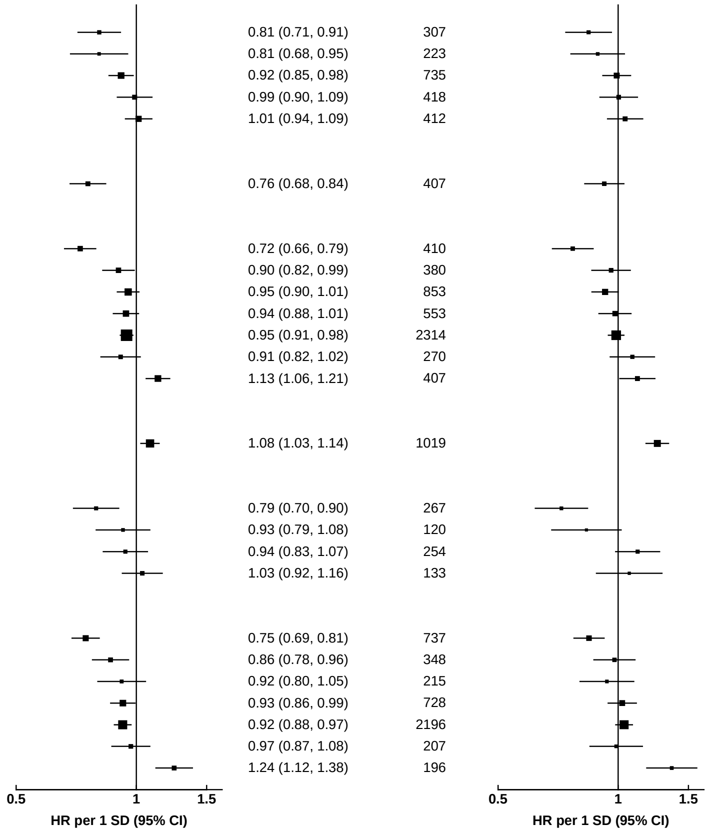

Condition	<4 years follow-up: Cases	<4 years follow-up: HR per 1 SD (95% CI)	4+ years follow-up: Cases	4+ years follow-up: HR per 1 SD (95% CI)	Phet
**Circulatory disease**					
Venous thromboembolism	366	0.81 (0.71, 0.91)	307	0.84 (0.74, 0.96)	0.64
Ischemic stroke	203	0.81 (0.68, 0.95)	223	0.89 (0.76, 1.04)	0.4
Ischemic heart disease	1007	0.92 (0.85, 0.98)	735	0.99 (0.92, 1.07)	0.14
Atrial fibrillation and flutter	480	0.99 (0.90, 1.09)	418	1.00 (0.90, 1.12)	0.86
Hemorrhoids	739	1.01 (0.94, 1.09)	412	1.04 (0.94, 1.15)	0.68
**Respiratory disease**					
Pneumonia	534	0.76 (0.68, 0.84)	407	0.92 (0.82, 1.03)	0.01
**Digestive disease**					
Gallbladder disease	698	0.72 (0.66, 0.79)	410	0.77 (0.68, 0.87)	0.41
Gastro-esophageal reflux disease	589	0.90 (0.82, 0.99)	380	0.96 (0.86, 1.07)	0.39
Diverticular disease	1243	0.95 (0.90, 1.01)	853	0.93 (0.86, 1.00)	0.57
Gastritis and duodenitis	885	0.94 (0.88, 1.01)	553	0.98 (0.90, 1.08)	0.49
Colon polyps	3536	0.95 (0.91, 0.98)	2314	0.99 (0.95, 1.03)	0.13
Non-infective enteritis and colitis	370	0.91 (0.82, 1.02)	270	1.09 (0.96, 1.23)	0.05
Inguinal hernia	856	1.13 (1.06, 1.21)	407	1.12 (1.01, 1.24)	0.83
**Joint disorder**					
Osteoarthritis	1683	1.08 (1.03, 1.14)	1019	1.25 (1.17, 1.34)	6e-04
**Genitourinary disease**					
Urinary tract infection	318	0.79 (0.70, 0.90)	267	0.72 (0.62, 0.84)	0.35
Kidney stones	189	0.93 (0.79, 1.08)	120	0.83 (0.68, 1.02)	0.41
Enlarged prostate	288	0.94 (0.83, 1.07)	254	1.12 (0.99, 1.27)	0.06
Female genital prolapse	372	1.03 (0.92, 1.16)	133	1.07 (0.88, 1.29)	0.79
**Other diseases**					
Diabetes	941	0.75 (0.69, 0.81)	737	0.85 (0.78, 0.92)	0.04
Iron deficiency anemia	456	0.86 (0.78, 0.96)	348	0.98 (0.87, 1.10)	0.11
Cellulitis	255	0.92 (0.80, 1.05)	215	0.94 (0.80, 1.09)	0.85
Non melanoma skin cancer	1006	0.93 (0.86, 0.99)	728	1.02 (0.94, 1.11)	0.06
Cataracts	2329	0.92 (0.88, 0.97)	2196	1.03 (0.99, 1.09)	0.001
Benign lesions of the uterus	365	0.97 (0.87, 1.08)	207	0.99 (0.85, 1.15)	0.83
Carpal tunnel syndrome	380	1.24 (1.12, 1.38)	196	1.36 (1.18, 1.57)	0.32

HRs and 95% CIs were estimated using Cox regression with age as the underlying time variable. Models were stratified by age group (< 50, 50–54, 55–59, 60–64, 65-70, and ≥ 70 years), and sex and adjusted for self-reported racial/ethnic group (White, other), socioeconomic status (Townsend index, fifths), education level (College or university degree/vocational qualification, further education, school leaver), employment (paid/self-employment, not employment, retired), smoking status (never, previous, current moderate (<15 cigarettes per day), current heavy (≥15 cigarettes per day), current unknown), alcohol consumption frequency (never, <3, 3+ /wk), BMI (<25, 25.0-29.9, 30.0-34.9, 35+ kg/m^2), and for females: HRT use (current, former, never), oral contraceptive pill (current, former, never), menopause status (yes, no), parity (none, 1-2, 3+). Heterogeneity in the associations for follow-up time was examined using in two different subgroups defined by follow-up period, using a $\chi 2$ for heterogeneity. The boxes represent the HRs, and vertical lines represent 95% CIs.
Abbreviations: CI=confidence interval; HR=hazard ratio; HRT= hormone replacement therapy.

© 2023 Watts EL et al. *JAMA Network Open*.

eFigure 6. Associations of Mean Accelerometer-Measured Physical Activity (Milligravity Units) With Risks of 25 Common Conditions Stratified by Age at Diagnosis

Condition	<68 years at diagnosis: Cases	<68 years at diagnosis: HR per 1 SD (95% CI)	68+ years at diagnosis: Cases	68+ years at diagnosis: HR per 1 SD (95% CI)	Phet
**Circulatory disease**					
Venous thromboembolism	305	0.85 (0.75, 0.96)	368	0.80 (0.70, 0.91)	0.54
Ischemic stroke	129	0.89 (0.74, 1.07)	297	0.83 (0.72, 0.95)	0.52
Ischemic heart disease	761	0.95 (0.88, 1.02)	981	0.94 (0.88, 1.02)	0.94
Atrial fibrillation and flutter	329	0.93 (0.83, 1.04)	569	1.04 (0.95, 1.15)	0.14
Hemorrhoids	739	1.04 (0.97, 1.12)	412	0.98 (0.87, 1.09)	0.36
**Respiratory disease**					
Pneumonia	308	0.93 (0.82, 1.04)	633	0.76 (0.69, 0.84)	0.01
**Digestive disease**					
Gallbladder disease	617	0.71 (0.64, 0.77)	491	0.80 (0.71, 0.89)	0.1
Gastro-esophageal reflux disease	545	0.93 (0.85, 1.01)	424	0.92 (0.82, 1.03)	0.87
Diverticular disease	1076	0.98 (0.92, 1.04)	1020	0.89 (0.83, 0.96)	0.05
Gastritis and duodenitis	742	0.93 (0.86, 1.00)	696	0.99 (0.91, 1.08)	0.3
Colon polyps	3175	0.96 (0.93, 1.00)	2675	0.96 (0.92, 1.00)	0.85
Non-infective enteritis and colitis	346	0.95 (0.85, 1.06)	294	1.03 (0.90, 1.18)	0.38
Inguinal hernia	561	1.17 (1.09, 1.27)	702	1.08 (0.99, 1.17)	0.14
**Joint disorder**					
Osteoarthritis	1238	1.13 (1.07, 1.20)	1464	1.16 (1.09, 1.23)	0.54
**Genitourinary disease**					
Urinary tract infection	218	0.78 (0.68, 0.91)	367	0.74 (0.65, 0.85)	0.58
Kidney stones	193	0.91 (0.79, 1.06)	116	0.84 (0.67, 1.05)	0.51
Enlarged prostate	204	1.00 (0.87, 1.15)	338	1.04 (0.93, 1.18)	0.66
Female genital prolapse	280	1.03 (0.91, 1.17)	225	1.05 (0.90, 1.23)	0.82
**Other diseases**					
Diabetes	746	0.78 (0.72, 0.85)	932	0.79 (0.73, 0.86)	0.85
Iron deficiency anemia	378	0.92 (0.83, 1.02)	426	0.89 (0.80, 1.01)	0.71
Cellulitis	236	0.96 (0.84, 1.10)	234	0.88 (0.75, 1.03)	0.38
Non melanoma skin cancer	624	0.99 (0.91, 1.07)	1110	0.95 (0.88, 1.02)	0.44
Cataracts	1126	1.00 (0.94, 1.07)	3399	0.96 (0.93, 1.00)	0.3
Benign lesions of the uterus	445	0.97 (0.88, 1.07)	127	0.99 (0.80, 1.22)	0.92
Carpal tunnel syndrome	292	1.26 (1.12, 1.40)	284	1.32 (1.16, 1.51)	0.57

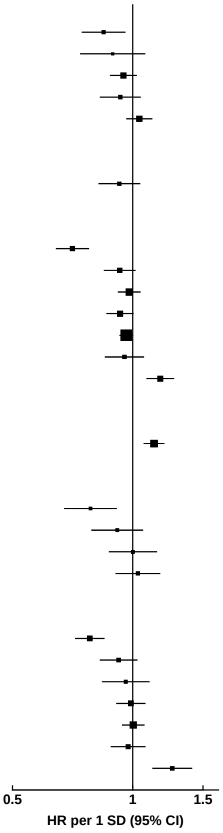

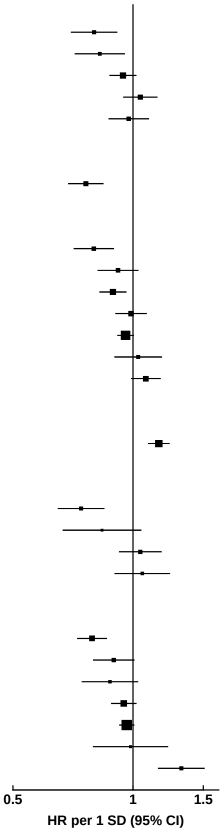

HRs and 95% CIs were estimated using Cox regression with age as the underlying time variable. Models were stratified by age group (< 50, 50–54, 55–59, 60–64, 65-70, and ≥ 70 years), and sex and adjusted for self-reported racial/ethnic group (White, other), socioeconomic status (Townsend index, fifths), education level (College or university degree/vocational qualification, further education, school leaver), employment (paid/self-employment, not employment, retired), smoking status (never, previous, current moderate (<15 cigarettes per day), current heavy (≥15 cigarettes per day), current unknown), alcohol consumption frequency (never, <3, 3+ /wk), BMI (<25, 25.0-29.9, 30.0-34.9, 35+ kg/m^2), and for females: HRT use (current, former, never), oral contraceptive pill (current, former, never), menopause status (yes, no), parity (none, 1-2, 3+). Heterogeneity in the associations for follow-up time was examined using in two different subgroups defined by follow-up period, using a $\chi 2$ for heterogeneity. The boxes represent the HRs, and vertical lines represent 95% CIs.

Abbreviations: CI=confidence interval; HR=hazard ratio; HRT= hormone replacement therapy.

© 2023 Watts EL et al. *JAMA Network Open*.

eFigure 7. Associations of Accelerometer-Measured Physical Activity (Milligravity Units) With Risks of 25 Common Conditions Stratified by Sex

Condition	Females: Cases	Females: HR per 1 SD (95% CI)	Males: Cases	Males: HR per 1 SD (95% CI)	Phet
**Circulatory disease**					
Venous thromboembolism	313	0.79 (0.69, 0.91)	360	0.85 (0.75, 0.95)	0.48
Ischemic stroke	196	0.81 (0.68, 0.96)	230	0.88 (0.76, 1.02)	0.47
Ischemic heart disease	573	0.94 (0.85, 1.03)	1169	0.95 (0.89, 1.01)	0.79
Atrial fibrillation and flutter	376	0.93 (0.83, 1.05)	522	1.03 (0.94, 1.13)	0.19
Hemorrhoids	625	1.04 (0.95, 1.13)	526	1.01 (0.92, 1.10)	0.66
**Respiratory disease**					
Pneumonia	444	0.87 (0.78, 0.98)	497	0.79 (0.71, 0.88)	0.20
**Digestive disease**					
Gallbladder disease	701	0.75 (0.69, 0.82)	407	0.72 (0.64, 0.81)	0.60
Gastro-esophageal reflux disease	592	0.85 (0.78, 0.94)	377	1.02 (0.92, 1.13)	0.01
Diverticular disease	1185	0.98 (0.92, 1.04)	911	0.91 (0.84, 0.97)	0.12
Gastritis and duodenitis	877	0.97 (0.90, 1.05)	561	0.94 (0.86, 1.02)	0.53
Colon polyps	3192	0.95 (0.91, 0.99)	2658	0.98 (0.94, 1.02)	0.35
Non-infective enteritis and colitis	405	0.97 (0.87, 1.08)	235	1.01 (0.88, 1.15)	0.62
Inguinal hernia	109	1.18 (0.96, 1.45)	1154	1.12 (1.06, 1.19)	0.64
**Joint disorder**					
Osteoarthritis	1644	1.06 (1.00, 1.12)	1058	1.26 (1.19, 1.34)	<0.001
**Genitourinary disease**					
Urinary tract infection	287	0.83 (0.72, 0.96)	298	0.70 (0.61, 0.80)	0.08
Kidney stones	110	1.03 (0.84, 1.26)	199	0.83 (0.71, 0.96)	0.09
**Other diseases**					
Diabetes	677	0.85 (0.77, 0.93)	1001	0.75 (0.70, 0.81)	0.05
Iron deficiency anemia	470	0.92 (0.83, 1.03)	334	0.90 (0.80, 1.01)	0.70
Cellulitis	215	0.86 (0.74, 1.01)	255	0.97 (0.85, 1.11)	0.25
Non melanoma skin cancer	799	0.95 (0.87, 1.03)	935	0.98 (0.91, 1.05)	0.55
Cataracts	2739	1.00 (0.96, 1.05)	1786	0.94 (0.89, 0.99)	0.08
Carpal tunnel syndrome	365	1.27 (1.14, 1.42)	211	1.30 (1.14, 1.49)	0.76

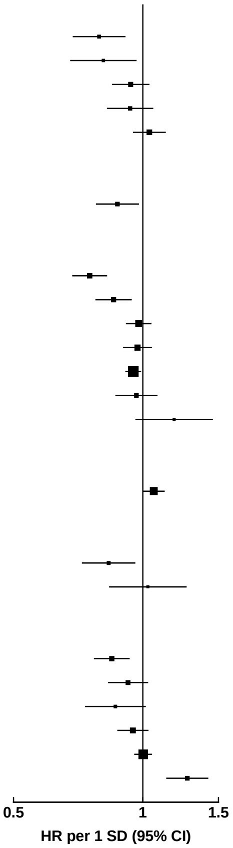

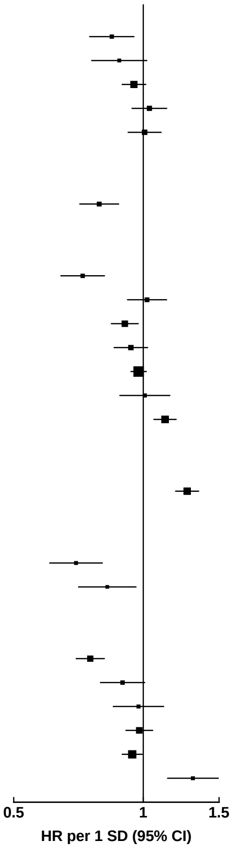

HRs and 95% CIs were estimated using Cox regression with age as the underlying time variable. Models were stratified by age group (< 50, 50–54, 55–59, 60–64, 65-70, and ≥ 70 years), and sex and adjusted for self-reported racial/ethnic group (White, other), socioeconomic status (Townsend index, fifths), education level (College or university degree/vocational qualification, further education, school leaver), employment (paid/self-employment, not employment, retired), smoking status (never, previous, current moderate (<15 cigarettes per day), current heavy (≥15 cigarettes per day), current unknown), alcohol consumption frequency (never, <3, 3+ /wk), BMI (<25, 25.0-29.9, 30.0-34.9, 35+ kg/m^2), and for females: HRT use (current, former, never), oral contraceptive pill (current, former, never), menopause status (yes, no), parity (none, 1-2, 3+). Heterogeneity in the associations for the non-case dependent subgroups was assessed using a $\chi 2$ interaction term. The boxes represent the HRs, and vertical lines represent 95% CIs.

Abbreviations: CI=confidence interval; HR=hazard ratio; HRT= hormone replacement therapy; SD=standard deviation.

© 2023 Watts EL et al. *JAMA Network Open*.

eFigure 8. Associations of Accelerometer-Measured Physical Activity (Milligravity Units) With Risks of 25 Common Conditions Stratified by Obesity Status

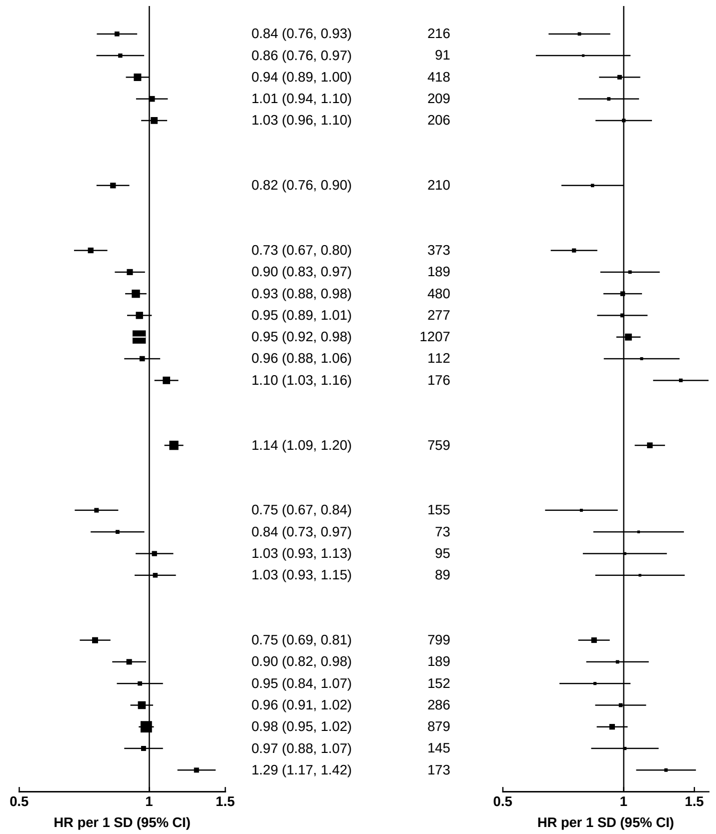

Condition	Cases (BMI <30 kg/m²)	HR per 1 SD (95% CI) (BMI <30 kg/m²)	Cases (BMI 30+ kg/m²)	HR per 1 SD (95% CI) (BMI 30+ kg/m²)	Phet
**Circulatory disease**					
Venous thromboembolism	457	0.84 (0.76, 0.93)	216	0.78 (0.65, 0.92)	0.43
Ischemic stroke	335	0.86 (0.76, 0.97)	91	0.79 (0.61, 1.04)	0.61
Ischemic heart disease	1324	0.94 (0.89, 1.00)	418	0.98 (0.87, 1.10)	0.55
Atrial fibrillation and flutter	689	1.01 (0.94, 1.10)	209	0.92 (0.77, 1.09)	0.3
Hemorrhoids	945	1.03 (0.96, 1.10)	206	1.00 (0.85, 1.17)	0.77
**Respiratory disease**					
Pneumonia	731	0.82 (0.76, 0.90)	210	0.84 (0.70, 1.00)	0.88
**Digestive disease**					
Gallbladder disease	735	0.73 (0.67, 0.80)	373	0.75 (0.66, 0.86)	0.73
Gastro-esophageal reflux disease	780	0.90 (0.83, 0.97)	189	1.04 (0.88, 1.23)	0.14
Diverticular disease	1616	0.93 (0.88, 0.98)	480	0.99 (0.89, 1.11)	0.28
Gastritis and duodenitis	1161	0.95 (0.89, 1.01)	277	0.99 (0.86, 1.14)	0.57
Colon polyps	4643	0.95 (0.92, 0.98)	1207	1.03 (0.96, 1.10)	0.03
Non-infective enteritis and colitis	528	0.96 (0.88, 1.06)	112	1.11 (0.90, 1.37)	0.24
Inguinal hernia	1087	1.10 (1.03, 1.16)	176	1.39 (1.19, 1.62)	0.006
**Joint disorder**					
Osteoarthritis	1943	1.14 (1.09, 1.20)	759	1.16 (1.07, 1.26)	0.7
**Genitourinary disease**					
Urinary tract infection	430	0.75 (0.67, 0.84)	155	0.78 (0.64, 0.96)	0.75
Kidney stones	236	0.84 (0.73, 0.97)	73	1.09 (0.84, 1.41)	0.09
Enlarged prostate	447	1.03 (0.93, 1.13)	95	1.01 (0.79, 1.28)	0.87
Female genital prolapse	416	1.03 (0.93, 1.15)	89	1.10 (0.85, 1.41)	0.66
**Other diseases**					
Diabetes	879	0.75 (0.69, 0.81)	799	0.84 (0.77, 0.92)	0.05
Iron deficiency anemia	615	0.90 (0.82, 0.98)	189	0.97 (0.81, 1.15)	0.47
Cellulitis	318	0.95 (0.84, 1.07)	152	0.85 (0.69, 1.04)	0.33
Non melanoma skin cancer	1448	0.96 (0.91, 1.02)	286	0.98 (0.85, 1.13)	0.77
Cataracts	3646	0.98 (0.95, 1.02)	879	0.94 (0.86, 1.02)	0.29
Benign lesions of the uterus	427	0.97 (0.88, 1.07)	145	1.01 (0.83, 1.22)	0.74
Carpal tunnel syndrome	403	1.29 (1.17, 1.42)	173	1.27 (1.08, 1.51)	0.92

HRs and 95% CIs were estimated using Cox regression with age as the underlying time variable. Models were stratified by age group (< 50, 50–54, 55–59, 60–64, 65-70, and ≥ 70 years), and sex and adjusted for self-reported racial/ethnic group (White, other), socioeconomic status (Townsend index, fifths), education level (College or university degree/vocational qualification, further education, school leaver), employment (paid/self-employment, not employment, retired), smoking status (never, previous, current moderate (<15 cigarettes per day), current heavy (≥15 cigarettes per day), current unknown), alcohol consumption frequency (never, <3, 3+ /wk), BMI (<25, 25.0-29.9, 30.0-34.9, 35+ kg/m^2), and for females: HRT use (current, former, never), oral contraceptive pill (current, former, never), menopause status (yes, no), parity (none, 1-2, 3+). Heterogeneity in the associations for the non-case dependent subgroups was assessed using a $\chi 2$ interaction term. The boxes represent the HRs, and vertical lines represent 95% CIs.

Abbreviations: BMI=body mass index; CI=confidence interval; HR=hazard ratio; HRT= hormone replacement therapy; SD=standard deviation.

© 2023 Watts EL et al. *JAMA Network Open*.

eFigure 9. Associations of Accelerometer-Measured Physical Activity (Milligravity Units) With Risks of 25 Common Conditions Stratified by Smoking Status

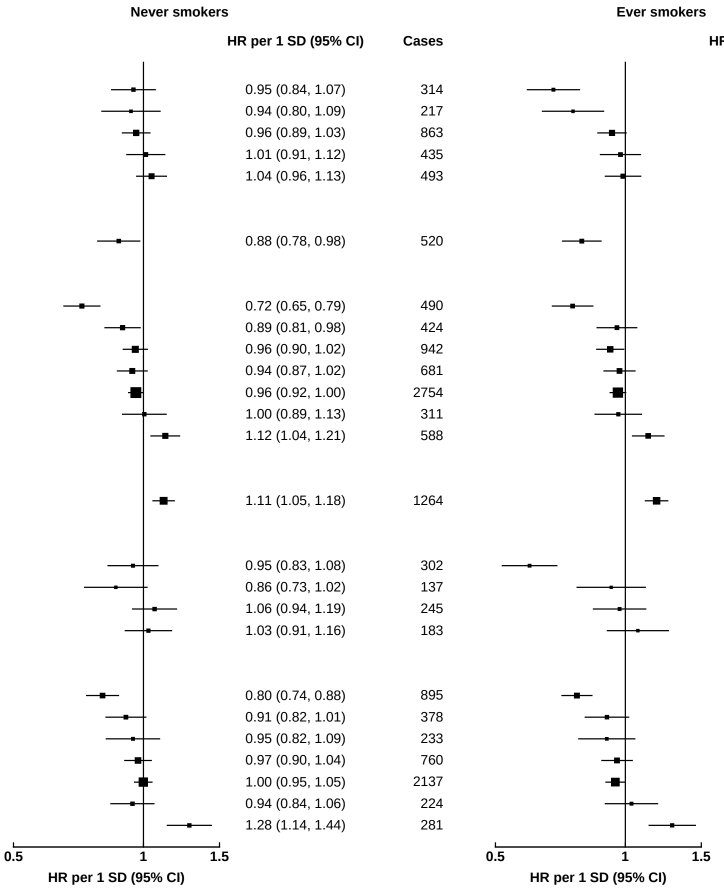

Condition	Never smokers: Cases	Never smokers: HR per 1 SD (95% CI)	Ever smokers: Cases	Ever smokers: HR per 1 SD (95% CI)	Phet
**Circulatory disease**					
Venous thromboembolism	358	0.95 (0.84, 1.07)	314	0.68 (0.59, 0.78)	0.0004
Ischemic stroke	208	0.94 (0.80, 1.09)	217	0.76 (0.64, 0.89)	0.06
Ischemic heart disease	874	0.96 (0.89, 1.03)	863	0.93 (0.86, 1.00)	0.55
Atrial fibrillation and flutter	462	1.01 (0.91, 1.12)	435	0.97 (0.88, 1.08)	0.62
Hemorrhoids	657	1.04 (0.96, 1.13)	493	0.99 (0.90, 1.09)	0.38
**Respiratory disease**					
Pneumonia	416	0.88 (0.78, 0.98)	520	0.79 (0.72, 0.88)	0.20
**Digestive disease**					
Gallbladder disease	614	0.72 (0.65, 0.79)	490	0.76 (0.68, 0.84)	0.52
Gastro-esophageal reflux disease	542	0.89 (0.81, 0.98)	424	0.96 (0.86, 1.06)	0.35
Diverticular disease	1149	0.96 (0.90, 1.02)	942	0.92 (0.86, 0.99)	0.46
Gastritis and duodenitis	753	0.94 (0.87, 1.02)	681	0.97 (0.89, 1.05)	0.62
Colon polyps	3085	0.96 (0.92, 1.00)	2754	0.96 (0.92, 1.00)	0.97
Non-infective enteritis and colitis	329	1.00 (0.89, 1.13)	311	0.96 (0.85, 1.09)	0.63
Inguinal hernia	675	1.12 (1.04, 1.21)	588	1.13 (1.04, 1.23)	0.92
**Joint disorder**					
Osteoarthritis	1429	1.11 (1.05, 1.18)	1264	1.18 (1.11, 1.25)	0.16
**Genitourinary disease**					
Urinary tract infection	282	0.95 (0.83, 1.08)	302	0.60 (0.52, 0.69)	<0.001
Kidney stones	171	0.86 (0.73, 1.02)	137	0.93 (0.77, 1.11)	0.57
Enlarged prostate	297	1.06 (0.94, 1.19)	245	0.97 (0.84, 1.12)	0.34
Female genital prolapse	321	1.03 (0.91, 1.16)	183	1.07 (0.91, 1.26)	0.69
**Other diseases**					
Diabetes	778	0.80 (0.74, 0.88)	895	0.77 (0.71, 0.84)	0.50
Iron deficiency anemia	423	0.91 (0.82, 1.01)	378	0.91 (0.81, 1.02)	0.96
Cellulitis	237	0.95 (0.82, 1.09)	233	0.91 (0.78, 1.05)	0.69
Non melanoma skin cancer	967	0.97 (0.90, 1.04)	760	0.96 (0.88, 1.04)	0.79
Cataracts	2374	1.00 (0.95, 1.05)	2137	0.95 (0.90, 1.00)	0.13
Benign lesions of the uterus	348	0.94 (0.84, 1.06)	224	1.03 (0.90, 1.19)	0.32
Carpal tunnel syndrome	295	1.28 (1.14, 1.44)	281	1.28 (1.14, 1.45)	0.95

HRs and 95% CIs were estimated using Cox regression with age as the underlying time variable. Models were stratified by age group (< 50, 50–54, 55–59, 60–64, 65-70, and ≥ 70 years), and sex and adjusted for self-reported racial/ethnic group (White, other), socioeconomic status (Townsend index, fifths), education level (College or university degree/vocational qualification, further education, school leaver), employment (paid/self-employment, not employment, retired), smoking status (never, previous, current moderate (<15 cigarettes per day), current heavy (≥15 cigarettes per day), current unknown), alcohol consumption frequency (never, <3, 3+ /wk), BMI (<25, 25.0-29.9, 30.0-34.9, 35+ kg/m^2), and for females: HRT use (current, former, never), oral contraceptive pill (current, former, never), menopause status (yes, no), parity (none, 1-2, 3+). Heterogeneity in the associations for the non-case dependent subgroups was assessed using a $\chi 2$ interaction term. The boxes represent the HRs, and vertical lines represent 95% CIs.

Abbreviations: CI=confidence interval; HR=hazard ratio; HRT= hormone replacement therapy; SD=standard deviation.

© 2023 Watts EL et al. *JAMA Network Open*.

eFigure 10. Associations of Accelerometer-Measured Physical Activity (Milligravity Units) With Risks of 25 Common Conditions Stratified by Whether Job Requires Manual Labor

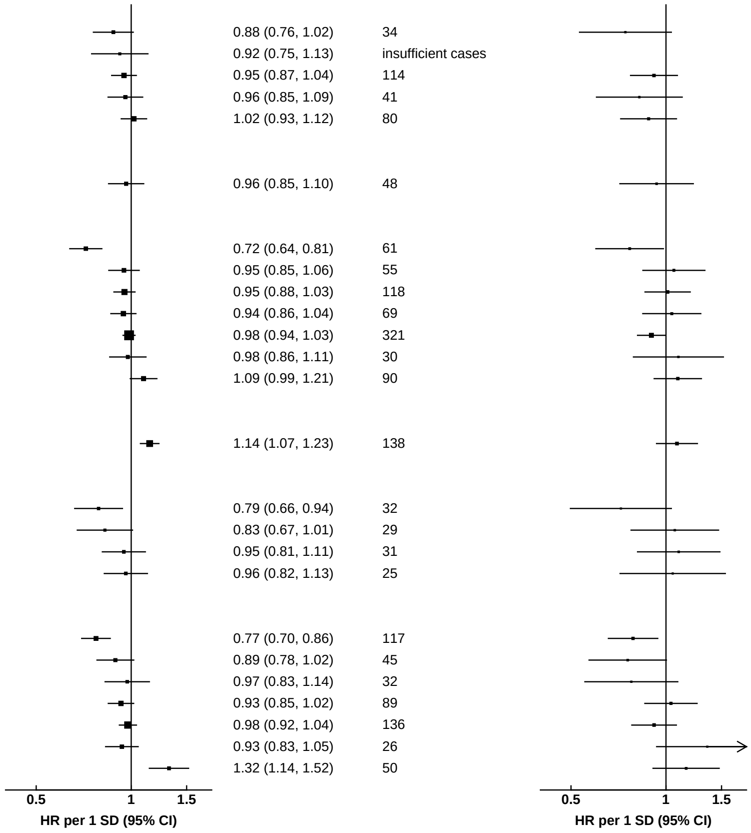

Condition	No manual labour: Cases	No manual labour: HR per 1 SD (95% CI)	Some manual labour: Cases	Some manual labour: HR per 1 SD (95% CI)	Phet
**Circulatory disease**					
Venous thromboembolism	252	0.88 (0.76, 1.02)	34	0.74 (0.53, 1.04)	0.37
Ischemic stroke	123	0.92 (0.75, 1.13)	insufficient cases		
Ischemic heart disease	624	0.95 (0.87, 1.04)	114	0.92 (0.77, 1.09)	0.72
Atrial fibrillation and flutter	316	0.96 (0.85, 1.09)	41	0.82 (0.60, 1.13)	0.38
Hemorrhoids	521	1.02 (0.93, 1.12)	80	0.88 (0.72, 1.08)	0.2
**Respiratory disease**					
Pneumonia	304	0.96 (0.85, 1.10)	48	0.93 (0.71, 1.22)	0.83
**Digestive disease**					
Gallbladder disease	436	0.72 (0.64, 0.81)	61	0.77 (0.60, 0.98)	0.64
Gastro-esophageal reflux disease	401	0.95 (0.85, 1.06)	55	1.06 (0.84, 1.33)	0.39
Diverticular disease	847	0.95 (0.88, 1.03)	118	1.01 (0.86, 1.19)	0.51
Gastritis and duodenitis	589	0.94 (0.86, 1.04)	69	1.04 (0.84, 1.29)	0.4
Colon polyps	2492	0.98 (0.94, 1.03)	321	0.90 (0.81, 1.00)	0.11
Non-infective enteritis and colitis	271	0.98 (0.86, 1.11)	30	1.10 (0.79, 1.52)	0.52
Inguinal hernia	462	1.09 (0.99, 1.21)	90	1.09 (0.92, 1.30)	0.97
**Joint disorder**					
Osteoarthritis	1024	1.14 (1.07, 1.23)	138	1.08 (0.93, 1.26)	0.51
**Genitourinary disease**					
Urinary tract infection	185	0.79 (0.66, 0.94)	32	0.72 (0.50, 1.04)	0.66
Kidney stones	126	0.83 (0.67, 1.01)	29	1.07 (0.78, 1.47)	0.18
Enlarged prostate	196	0.95 (0.81, 1.11)	31	1.10 (0.81, 1.48)	0.4
Female genital prolapse	204	0.96 (0.82, 1.13)	25	1.05 (0.72, 1.54)	0.68
**Other diseases**					
Diabetes	563	0.77 (0.70, 0.86)	117	0.79 (0.66, 0.94)	0.88
Iron deficiency anemia	283	0.89 (0.78, 1.02)	45	0.76 (0.57, 1.00)	0.3
Cellulitis	194	0.97 (0.83, 1.14)	32	0.78 (0.55, 1.09)	0.24
Non melanoma skin cancer	592	0.93 (0.85, 1.02)	89	1.04 (0.86, 1.26)	0.3
Cataracts	1339	0.98 (0.92, 1.04)	136	0.92 (0.78, 1.08)	0.48
Benign lesions of the uterus	341	0.93 (0.83, 1.05)	26	1.35 (0.93, 1.96)	0.06
Carpal tunnel syndrome	205	1.32 (1.14, 1.52)	50	1.16 (0.91, 1.47)	0.37

HRs and 95% CIs were estimated using Cox regression with age as the underlying time variable. Models were stratified by age group (< 50, 50–54, 55–59, 60–64, 65-70, and ≥ 70 years), and sex and adjusted for self-reported racial/ethnic group (White, other), socioeconomic status (Townsend index, fifths), education level (College or university degree/vocational qualification, further education, school leaver), employment (paid/self-employment, not employment, retired), smoking status (never, previous, current moderate (<15 cigarettes per day), current heavy (≥15 cigarettes per day), current unknown), alcohol consumption frequency (never, <3, 3+ /wk), BMI (<25, 25.0-29.9, 30.0-34.9, 35+ kg/m^2), and for females: HRT use (current, former, never), oral contraceptive pill (current, former, never), menopause status (yes, no), parity (none, 1-2, 3+). Heterogeneity in the associations for the non-case dependent subgroups was assessed using a $\chi 2$ interaction term. The boxes represent the HRs, and vertical lines represent 95% CIs.

Abbreviations: CI=confidence interval; HR=hazard ratio; HRT= hormone replacement therapy; SD=standard deviation.

© 2023 Watts EL et al. *JAMA Network Open*.

eTable 1. Disease Outcome Definitions and Exclusion Criteria

**Admission cause**	**Outcome definition using ICD-10***	**Relevant procedure code using OPSC-4 definition†**	**Exclusion criteria using ICD-10***	**Exclusion criteria using OPSC-4**	**Exclusion criteria using ICD-9**	**Exclusion criteria using touchscreen†**	**Exclusion criteria using UKB interviews for outcomes***	**Exclusion criteria using cancer registry‡**
Ischemic heart diseases	I20, I21, I22, I23, I24, I25		I48, I20, I21, I22, I23, I24, I25, G45, I60, I61, I62, I63, I64, I65, I66, I67, I68, I69, E10, E11, E13, E14		427.31, 427.32, 410, 411, 412, 413, 414, 430, 431, 432, 433, 434, 435, 436, 437, 438, 250	UKB variable 6150: 'Has a doctor ever told you that you have the following conditions?'<br>1) heart attack<br>2) angina<br>3) stroke	1471 atrial fibrillation<br>1483 atrial flutter<br>1074 angina<br>1075 heart attack/myocardial infarction<br>1081 stroke<br>1082 transient ischemic attack<br>1083 subdural hemorrhage/hematoma<br>1086 subarachnoid hemorrhage	
Atrial fibrillation and flutter	I48		I48, I20, I21, I22, I23, I24, I25, G45, I60, I61, I62, I63, I64, I65, I66, I67, I68, I69, E10, E11, E13, E14		427.31, 427.32, 410, 411, 412, 413, 414, 430, 431, 432, 433, 434, 435, 436, 437, 438, 250	UKB variable 6150: 'Has a doctor ever told you that you have the following conditions?'<br>1) heart attack<br>2) angina<br>3) stroke	1471 atrial fibrillation<br>1483 atrial flutter<br>1074 angina<br>1075 heart attack/myocardial infarction<br>1081 stroke<br>1082 transient ischemic attack<br>1083 subdural hemorrhage/hematoma<br>1086 subarachnoid hemorrhage	
Ischemic stroke	I63		I48, I20, I21, I22, I23, I24, I25, G45, I60, I61, I62, I63, I64, I65, I66, I67, I68, I69, E10, E11, E13, E14		427.31, 427.32, 410, 411, 412, 413, 414, 430, 431, 432, 433, 434, 435, 436, 437, 438, 250	UKB variable 6150: 'Has a doctor ever told you that you have the following conditions?'<br>1) heart attack<br>2) angina<br>3) stroke	1471 atrial fibrillation<br>1483 atrial flutter<br>1074 angina<br>1075 heart attack/myocardial infarction<br>1081 stroke<br>1082 transient ischemic attack<br>1083 subdural hemorrhage/hematoma<br>1086 subarachnoid hemorrhage	
Venous thromboembolism	I26, I80, 181, I82		I26, I80, 181, I82, E10, E11, E13, E14		415.1, 451, 452, 453, 250		1068 venous thromboembolic disease	

© 2023 Watts EL et al. *JAMA Network Open*.

							1094 deep venous thrombosis	
Hemorrhoids	I84	H51, H52, H53	I84, E10, E11, E13, E14	H51, H52, H53	455, 250		1505 hemorrhoids / piles	
Pneumonia	J18		J18, E10, E11, E13, E14		480, 481, 482, 483, 484, 485, 486, 250		1398 pneumonia	
Gastro-esophageal reflux disease	K21		K21, E10, E11, E13, E14		530.11, 530.81, 250		1138 gastro-esophageal reflux	
Gastritis and duodenitis	K29		K29, E10, E11, E13, E14		535, 250			
Inguinal hernia	K40	T19, T20, T21	K40, E10, E11, E13, E14	T19, T20, T21	550, 250		1513 inguinal hernia	
Noninfective enteritis and colitis	K50, K51, K52		K50, K51, K52, E10, E11, E13, E14		555, 556, 558, 250		1459 colitis/not Crohn's or ulcerative colitis<br>1462 Crohn's disease<br>1463 ulcerative colitis	
Diverticular disease of intestine	K57		K57, E10, E11, E13, E14		562, 250		1458 diverticular disease/diverticulitis	
Colon polyps	D12, K63.5	H20, H221, H23, H251, H26, H281	D12, K63.5, E10, E11, E13, E14	H20, H221, H23, H251, H26, H281	211.3, 211.4, 250		1460 rectal or colon adenoma/polyps	Cases diagnosed +/- 1 year of colorectal cancer diagnosis (C18-20)
Gallbladder disease	K80, K81	J18	K80, K81, E10, E11, E13, E14	J18	574, 575.0, 575.1, 250		1161 gallbladder disease	
Osteoarthritis	M15, M16, M17, M18, M19, M47		M15, M16, M17, M18, M19, M47, E10, E11, E13, E14		715, 721, 250		1465 osteoarthritis	
Kidney stones	N20, N23		N20, N23, E10, E11, E13, E14		592, 788.0, 250		1197 kidney stone/ureter stone/bladder stone	
Urinary tract infection	N39.0		N39.0, E10, E11, E13, E14		599.0, 250		1196 urinary tract infection/kidney infection	
Hyperplasia of prostate	N40		N40, E10, E11, E13, E14		600, 250		1516 bph / benign prostatic hypertrophy<br>1396 enlarged prostate	
Female genital prolapse	N81	M51, M52, M53, P22, P23, P24	N81, E10, E11, E13, E14	M51, M52, M53, P22, P23, P24	618, 250		1353 vaginal prolapse/uterine prolapse	
Benign neoplasms of uterus	D25 D26	Q181, Q171, Q172, Q173, Q174, Q092, Q093, Q094, Q161	D25, D26, E10, E11, E13, E14	Q181, Q171, Q172, Q173, Q174, Q07, Q08, Q092, Q093, Q094, Q161	218, 250	UKB variable 'Ever had hysterectomy 3591':1 yes,<br>UKB variable 'Ever had menopause 2724':2 don't know had hysterectomy	1351 uterine fibroids<br>1352 uterine polyps	Any previous cancer (excluding C44)<br>Cases diagnosed +/- 1 year of uterine cancer diagnosis (C53-55)
Iron deficiency anemia	D50		D50, E10, E11, E13, E14		280, 250		1330 iron deficiency anemia	

© 2023 Watts EL et al. *JAMA Network Open*.

Diabetes mellitus	E10, E11, E13, E14		E10, E11, E13, E14		250, 250		1220 diabetes<br>1221 gestational diabetes<br>1222 type 1 diabetes<br>1223 type 2 diabetes	
Carpal tunnel syndrome	G56.0	A65.1	G56.0, E10, E11, E13, E14	A65.1	354.0, 250		1541 carpal tunnel syndrome	
Cataract	H25, H26, Q120	C71, C72, C73, C74, C75	H25, H26, Q120, E10, E11, E13, E14	C71, C72, C73, C74, C75	366, 250	UKB variable 'Have eye problems 6148':4 Cataract	1278 cataract	
Cellulitis	L03		L03, E10, E11, E13, E14		681, 682, 250		1625 cellulitis	
Nonmelanoma skin cancer	C44		C44, E10, E11, E13, E14		173, 250			Any previous cancer
*Based on ICD-10 definition and UKB variable 20002<br>†All participants were also excluded using UKB variable 2443 ‘Has a doctor ever told you that you have diabetes?’ 1: yes; and UKB variable 6177 'Taking medications ' 3: insulin								
‡All participants with history of cancer from cancer registries (excluding C44) were excluded								

© 2023 Watts EL et al. *JAMA Network Open*.

eTable 2. Disease Definitions and 5-Year Case Fatality Rate*

Condition	ICD-10	Disease summary	5-year case fatality rate*, %
**Circulatory disease**			
Venous thromboembolism	I26 I80 181 I82	Blood clot that starts in a vein.	0.63
Ischemic stroke	I63	Interrupted or reduced blood supply to part of the brain.	1.63
Ischemic heart disease	I20 I21 I22 I23 I24 I25	Narrowed heart arteries, reducing blood and oxygen supply to the heart.	1.78
Atrial fibrillation and flutter	I48	Irregular and often rapid heart rhythm.	0.12
Hemorrhoids	I84 K64	Swellings containing enlarged blood vessels that are found inside or around the rectum and anus.	0.00
**Respiratory disease**			
Pneumonia	J18	Infection of the lungs.	1.48
**Digestive disease**			
Gallbladder disease	K80 K81	Inflammation, infection, stones, or blockage of the gallbladder	0.08
Gastro-esophageal reflux disease	K21	Stomach acid leaks up into the esophagus.	0.00
Diverticular disease of intestine	K57	Development of small pouches (diverticula) in the lining of the intestine.	0.08
Gastritis and duodenitis	K29	Inflammation of stomach lining and duodenitis, respectively.	0.00
Colon polyps	D12 K63.5	Excess growth on the lining of the colon.	0.00
Noninfective enteritis and colitis	K50 K51 K52	Inflammation of the small intestine and colon, respectively.	0.09
Inguinal hernia	K40	Tissue protruding through a weak spot in the abdominal muscles.	0.00
**Joint disorder**			
Osteoarthritis	M15 M16 M17 M18 M19 M47	The most common type of arthritis that occurs when tissue at the ends of bones wears down.	0.01
**Genitourinary disease**			
Urinary tract infection	N39.0	Infection in kidneys, bladder, ureters, or urethra.	0.14
Kidney stones	N20 N23	Small, hard deposits that form in the kidneys.	0.00
Hyperplasia of prostate	N40	Enlarged prostate gland.	0.00
Female genital prolapse	N81	Organs in the pelvis slip down from their normal position and bulge into the vagina.	0.00
**Other diseases**			
Diabetes mellitus	E10 E11 E12 E13 E14	The body does not produce enough or respond normally to insulin.	0.76
Iron deficiency anemia	D50	Iron deficiency leads to a reduction in cell blood supply.	0.00
Cellulitis	L03	Bacterial skin infection.	0.07
Nonmelanoma skin cancer	C44	All the types of skin cancers that are not melanoma.	0.08
Cataract	H25 H26 Q12.0	Development of a cloudy area in the lens of the eye that leads to a decrease in vision.	0.00
Benign neoplasms of the uterus	D25 D26	Noncancerous growths of the uterus.	0.02
Carpal tunnel syndrome	G56.0	Pressure on the median nerve.	0.00

*5-year case fatality rate = n deaths/n cases x 100, within a 5-year period. Calculated based on incident cases in the full UK Biobank population with no prior history of each respective disease, diabetes or cancer (in parallel with primary analysis), includes the primary cause of death only.

© 2023 Watts EL et al. *JAMA Network Open*.

eTable 3. Associations of Time Spent Doing Sedentary, Light, and Moderate to Vigorous Physical Activity With Risks of 25 Common Conditions

Condition	Model	Sedentary	Light	Moderate-to-vigorous
		HR per 20-min/day (95% CI)	HR per 20-min/day (95% CI)	HR per 20-min/day (95% CI)
Venous thromboembolism	1-factor model*	1.02 (1.00, 1.03)	**0.98 (0.96, 0.99)**	**0.93 (0.87, 0.99)**
	2-factor model†	1.00 (0.98, 1.03)	0.98 (0.96, 1.01)	-
	2-factor model†	-	**0.98 (0.96, 1.00)**	0.93 (0.87, 0.99)
	2-factor model†	1.01 (1.00, 1.03)	-	0.94 (0.88, 1.01)
	Partition model‡	0.99 (0.97, 1.02)	0.97 (0.95, 1.00)	0.92 (0.86, 0.99)
Ischemic stroke	1-factor model*	1.02 (1.00, 1.04)	0.98 (0.96, 1.00)	**0.89 (0.82, 0.97)**
	2-factor model†	1.01 (0.98, 1.04)	0.99 (0.96, 1.02)	-
	2-factor model†	-	0.98 (0.96, 1.00)	**0.89 (0.82, 0.97)**
	2-factor model†	1.01 (0.99, 1.03)	-	**0.90 (0.83, 0.99)**
	Partition model‡	1.00 (0.97, 1.03)	0.98 (0.95, 1.01)	**0.89 (0.81, 0.98)**
Ischemic heart disease	1-factor model*	1.01 (1.00, 1.02)	1.00 (0.99, 1.01)	**0.94 (0.90, 0.97)**
	2-factor model†	**1.02 (1.01, 1.04)**	**1.02 (1.01, 1.04)**	-
	2-factor model†	-	1.00 (0.99, 1.01)	**0.94 (0.90, 0.97)**
	2-factor model†	1.00 (0.99, 1.01)	-	**0.94 (0.90, 0.98)**
	Partition model‡	1.02 (1.00, 1.03)	1.02 (1.00, 1.03)	**0.95 (0.91, 0.99)**
Atrial fibrillation and flutter	1-factor model*	1.01 (1.00, 1.02)	1.00 (0.98, 1.01)	0.98 (0.93, 1.03)
	2-factor model†	1.02 (1.00, 1.04)	1.02 (1.00, 1.04)	-
	2-factor model†	-	1.00 (0.99, 1.01)	0.98 (0.93, 1.03)
	2-factor model†	1.01 (1.00, 1.02)	-	0.99 (0.93, 1.04)
	Partition model‡	1.02 (1.00, 1.04)	1.02 (0.99, 1.04)	1.00 (0.94, 1.06)
Hemorrhoids	1-factor model*	1.00 (0.98, 1.01)	1.00 (0.99, 1.02)	0.99 (0.95, 1.04)
	2-factor model†	1.00 (0.98, 1.01)	1.00 (0.98, 1.02)	-
	2-factor model†	-	1.00 (0.99, 1.02)	0.99 (0.95, 1.04)
	2-factor model†	1.00 (0.98, 1.01)	-	0.99 (0.94, 1.04)
	Partition model‡	0.99 (0.97, 1.01)	1.00 (0.98, 1.02)	0.99 (0.94, 1.04)
Pneumonia	1-factor model*	**1.02 (1.01, 1.04)**	0.99 (0.97, 1.00)	**0.86 (0.81, 0.91)**
	2-factor model†	**1.04 (1.02, 1.06)**	1.02 (0.99, 1.04)	-
	2-factor model†	-	0.99 (0.98, 1.00)	**0.86 (0.81, 0.92)**
	2-factor model†	**1.02 (1.00, 1.03)**	-	**0.87 (0.82, 0.93)**
	Partition model‡	1.02 (1.00, 1.04)	1.01 (0.98, 1.03)	**0.88 (0.82, 0.94)**
Gallbladder disease	1-factor model*	**1.03 (1.02, 1.04)**	**0.96 (0.95, 0.97)**	**0.79 (0.75, 0.85)**
	2-factor model†	1.00 (0.98, 1.01)	**0.96 (0.94, 0.98)**	-
	2-factor model†	-	**0.96 (0.95, 0.97)**	**0.80 (0.75, 0.85)**
	2-factor model†	**1.02 (1.01, 1.03)**	-	**0.81 (0.76, 0.86)**
	Partition model‡	**0.98 (0.96, 0.99)**	**0.94 (0.93, 0.96)**	**0.78 (0.74, 0.84)**
Diverticular disease	1-factor model*	1.00 (0.99, 1.01)	1.00 (0.99, 1.01)	**0.90 (0.86, 0.93)**
	2-factor model†	1.01 (1.00, 1.02)	1.01 (1.00, 1.02)	-
	2-factor model†	-	1.00 (0.99, 1.01)	**0.90 (0.86, 0.93)**
	2-factor model†	1.00 (0.99, 1.01)	-	**0.90 (0.86, 0.93)**
	Partition model‡	1.00 (0.98, 1.01)	1.00 (0.99, 1.02)	**0.90 (0.86, 0.93)**
Gastro-esophageal reflux disease	1-factor model*	1.00 (0.98, 1.01)	1.00 (0.99, 1.01)	**0.89 (0.84, 0.94)**
	2-factor model†	0.99 (0.97, 1.01)	1.00 (0.98, 1.02)	-
	2-factor model†	-	1.00 (0.99, 1.02)	**0.89 (0.84, 0.94)**
	2-factor model†	0.99 (0.98, 1.00)	-	**0.88 (0.83, 0.94)**
	Partition model‡	0.98 (0.96, 1.00)	0.98 (0.96, 1.01)	**0.87 (0.82, 0.93)**
Colon polyps	1-factor model*	1.00 (1.00, 1.01)	1.00 (1.00, 1.01)	**0.96 (0.94, 0.98)**
	2-factor model†	1.00 (1.00, 1.01)	1.00 (1.00, 1.01)	-

© 2023 Watts EL et al. *JAMA Network Open*.

	2-factor model†	-	1.00 (1.00, 1.01)	**0.96 (0.94, 0.98)**
	2-factor model†	1.00 (0.99, 1.00)	-	**0.96 (0.94, 0.98)**
	Partition model‡	1.00 (0.99, 1.01)	1.00 (0.99, 1.01)	**0.96 (0.94, 0.98)**
Gastritis and duodenitis	1-factor model*	0.99 (0.98, 1.00)	1.00 (0.99, 1.01)	**0.95 (0.91, 0.99)**
	2-factor model†	0.99 (0.97, 1.00)	0.99 (0.97, 1.01)	-
	2-factor model†	-	1.00 (0.99, 1.01)	**0.95 (0.91, 0.99)**
	2-factor model†	0.99 (0.98, 1.00)	-	**0.94 (0.90, 0.98)**
	Partition model‡	**0.98 (0.96, 0.99)**	0.98 (0.96, 1.00)	**0.93 (0.89, 0.97)**
Non-infective enteritis and colitis	1-factor model*	0.99 (0.98, 1.01)	1.00 (0.99, 1.02)	1.01 (0.94, 1.07)
	2-factor model†	0.99 (0.96, 1.01)	0.99 (0.97, 1.02)	-
	2-factor model†	-	1.00 (0.99, 1.02)	1.01 (0.94, 1.07)
	2-factor model†	0.99 (0.98, 1.01)	-	1.00 (0.94, 1.07)
	Partition model‡	0.98 (0.96, 1.01)	0.99 (0.96, 1.02)	0.99 (0.93, 1.06)
Inguinal hernia	1-factor model*	**0.97 (0.96, 0.98)**	**1.04 (1.03, 1.05)**	1.02 (0.98, 1.06)
	2-factor model†	1.01 (0.99, 1.03)	**1.05 (1.03, 1.07)**	-
	2-factor model†	-	**1.04 (1.03, 1.05)**	1.02 (0.98, 1.06)
	2-factor model†	**0.97 (0.96, 0.98)**	-	0.99 (0.95, 1.03)
	Partition model‡	1.01 (0.99, 1.03)	**1.05 (1.03, 1.07)**	1.03 (0.98, 1.07)
Osteoarthritis	1-factor model*	**0.99 (0.98, 0.99)**	**1.03 (1.02, 1.04)**	0.98 (0.95, 1.02)
	2-factor model†	**1.02 (1.00, 1.03)**	**1.04 (1.03, 1.05)**	-
	2-factor model†	-	**1.03 (1.02, 1.04)**	0.98 (0.95, 1.02)
	2-factor model†	**0.98 (0.98, 0.99)**	-	0.97 (0.94, 1.00)
	Partition model‡	**1.02 (1.00, 1.03)**	**1.04 (1.03, 1.05)**	1.00 (0.96, 1.03)
Urinary tract infection	1-factor model*	**1.02 (1.00, 1.04)**	**0.97 (0.96, 0.99)**	**0.78 (0.72, 0.85)**
	2-factor model†	1.01 (0.98, 1.03)	0.98 (0.95, 1.01)	-
	2-factor model†	-	**0.98 (0.96, 0.99)**	**0.79 (0.72, 0.86)**
	2-factor model†	1.01 (0.99, 1.03)	-	**0.79 (0.73, 0.86)**
	Partition model‡	0.98 (0.96, 1.01)	**0.96 (0.94, 0.99)**	**0.78 (0.71, 0.85)**
Kidney stones	1-factor model*	1.01 (0.99, 1.03)	0.98 (0.96, 1.01)	0.96 (0.87, 1.05)
	2-factor model†	1.00 (0.96, 1.03)	0.98 (0.95, 1.02)	-
	2-factor model†	-	0.98 (0.96, 1.01)	0.96 (0.87, 1.05)
	2-factor model†	1.01 (0.99, 1.03)	-	0.97 (0.88, 1.06)
	Partition model‡	0.99 (0.96, 1.03)	0.98 (0.94, 1.01)	0.95 (0.86, 1.05)
Enlarged prostate	1-factor model*	0.99 (0.98, 1.01)	1.02 (1.00, 1.04)	0.96 (0.90, 1.03)
	2-factor model†	1.01 (0.98, 1.03)	1.02 (1.00, 1.05)	-
	2-factor model†	-	1.02 (1.00, 1.04)	0.96 (0.90, 1.03)
	2-factor model†	0.99 (0.97, 1.01)	-	0.95 (0.89, 1.02)
	Partition model‡	1.00 (0.98, 1.03)	1.02 (0.99, 1.05)	0.97 (0.90, 1.03)
Female genital prolapse	1-factor model*	**0.97 (0.95, 0.99)**	**1.03 (1.01, 1.05)**	0.96 (0.88, 1.05)
	2-factor model†	0.99 (0.96, 1.02)	1.02 (0.99, 1.05)	-
	2-factor model†	-	**1.03 (1.01, 1.05)**	0.96 (0.88, 1.05)
	2-factor model†	**0.97 (0.95, 0.99)**	-	0.94 (0.86, 1.02)
	Partition model‡	0.98 (0.95, 1.01)	1.01 (0.98, 1.04)	0.94 (0.86, 1.03)
Diabetes	1-factor model*	**1.03 (1.02, 1.04)**	**0.98 (0.97, 0.99)**	**0.78 (0.74, 0.82)**
	2-factor model†	**1.04 (1.03, 1.06)**	**1.02 (1.00, 1.03)**	-
	2-factor model†	-	**0.99 (0.98, 1.00)**	**0.78 (0.74, 0.82)**
	2-factor model†	**1.02 (1.01, 1.03)**	-	**0.79 (0.75, 0.83)**
	Partition model‡	**1.02 (1.01, 1.04)**	1.01 (0.99, 1.02)	**0.79 (0.75, 0.84)**
Cellulitis	1-factor model*	1.01 (0.99, 1.03)	1.02 (1.00, 1.04)	0.95 (0.88, 1.03)
	2-factor model†	**1.06 (1.03, 1.09)**	**1.07 (1.04, 1.11)**	-
	2-factor model†	-	1.02 (1.00, 1.04)	0.95 (0.88, 1.03)
	2-factor model†	1.01 (0.99, 1.03)	-	0.96 (0.88, 1.04)

© 2023 Watts EL et al. *JAMA Network Open*.

	Partition model‡	**1.06 (1.03, 1.09)**	**1.07 (1.04, 1.11)**	1.00 (0.92, 1.09)
Iron deficiency anemia	1-factor model*	**1.02 (1.00, 1.03)**	1.00 (0.98, 1.01)	**0.91 (0.85, 0.97)**
	2-factor model†	**1.03 (1.01, 1.06)**	1.03 (1.00, 1.05)	-
	2-factor model†	-	1.00 (0.98, 1.01)	**0.91 (0.85, 0.97)**
	2-factor model†	1.01 (1.00, 1.03)	-	**0.92 (0.86, 0.98)**
	Partition model‡	**1.03 (1.00, 1.05)**	1.02 (1.00, 1.04)	0.93 (0.87, 0.99)
Uterine fibroids	1-factor model*	1.00 (0.99, 1.02)	0.99 (0.98, 1.01)	0.93 (0.86, 1.01)
	2-factor model†	0.99 (0.97, 1.02)	0.99 (0.96, 1.02)	-
	2-factor model†	-	0.99 (0.98, 1.01)	0.93 (0.86, 1.01)
	2-factor model†	1.00 (0.98, 1.02)	-	0.93 (0.86, 1.01)
	Partition model‡	0.98 (0.96, 1.01)	0.98 (0.95, 1.01)	0.92 (0.85, 1.00)
Nonmelanoma skin cancer	1-factor model*	1.00 (0.99, 1.01)	1.00 (0.99, 1.01)	1.01 (0.97, 1.05)
	2-factor model†	0.99 (0.97, 1.00)	0.99 (0.97, 1.01)	-
	2-factor model†	-	1.00 (0.99, 1.01)	1.01 (0.97, 1.05)
	2-factor model†	1.00 (0.99, 1.01)	-	1.01 (0.97, 1.05)
	Partition model‡	0.99 (0.97, 1.00)	0.99 (0.97, 1.01)	1.00 (0.96, 1.04)
Cataracts	1-factor model*	**1.01 (1.00, 1.01)**	1.00 (0.99, 1.00)	**0.95 (0.93, 0.98)**
	2-factor model†	**1.01 (1.00, 1.02)**	1.01 (1.00, 1.02)	-
	2-factor model†	-	1.00 (0.99, 1.00)	**0.95 (0.93, 0.98)**
	2-factor model†	1.01 (1.00, 1.01)	-	**0.96 (0.93, 0.99)**
	Partition model‡	1.01 (1.00, 1.02)	1.00 (0.99, 1.01)	**0.96 (0.93, 0.99)**
Carpal tunnel syndrome	1-factor model*	0.99 (0.97, 1.00)	**1.04 (1.02, 1.06)**	0.94 (0.87, 1.01)
	2-factor model†	**1.05 (1.02, 1.07)**	**1.08 (1.05, 1.11)**	-
	2-factor model†	-	**1.04 (1.02, 1.06)**	0.93 (0.87, 1.01)
	2-factor model†	0.98 (0.97, 1.00)	-	0.92 (0.85, 1.00)
	Partition model‡	**1.04 (1.01, 1.07)**	**1.08 (1.05, 1.11)**	0.97 (0.89, 1.05)

HRs and 95% CIs were estimated using Cox regression with age as the underlying time variable. Models were stratified by age group (< 50, 50–54, 55–59, 60–64, 65-70, and ≥ 70 years), and sex and adjusted for self-reported racial/ethnic group (White, other), socioeconomic status (Townsend index, fifths), education level (College or university degree/vocational qualification, further education, school leaver), employment (paid/self-employment, not employment, retired), smoking status (never, previous, current moderate (<15 cigarettes per day), current heavy (≥15 cigarettes per day), current unknown), alcohol consumption frequency (never, <3, 3+ /wk), BMI (<25, 25.0-29.9, 30.0-34.9, 35+ kg/m^2), and for females: HRT use (current, former, never), oral contraceptive pill (current, former, never), menopause status (yes, no), parity (none, 1-2, 3+). Risk estimates are bold where p<0.03.

*Associations from separate models for each type of behavior, adjusted only for covariates.
†Associations from separate models that included two physical activity exposures (e.g., HR = sedentary time + light activity time + covariates).
‡ Associations from a single model that included sedentary time, light and moderate-to-vigorous activity, and covariates.

Abbreviations: CI=confidence interval; HR=hazard ratio; HRT= hormone replacement therapy.

© 2023 Watts EL et al. *JAMA Network Open*.

eTable 4. Estimated Percentage of Hospitalizations Potentially Prevented by Increasing Moderate to Vigorous Physical Activity by 20 Minutes per Day

Condition	Percentage reductions in hospitalizations following 20 min/day increase in MVPA, (95% CI)
**Circulatory disease**	
Venous thromboembolism	4.0 (-3.8, 11.9)
Ischemic heart disease	5.4 (-0.5, 11.3)
Ischemic stroke	9.0 (-0.8, 18.8)
Atrial fibrillation and flutter	-0.5 (-6.4, 5.3)
Hemorrhoids	3.2 (-2.7, 9.1)
**Respiratory disease**	
Pneumonia	**14.1 (8.3, 20.0)**
**Digestive disease**	
Gallbladder disease	**19.8 (14.0, 25.7)**
Gastro-esophageal reflux disease	**9.2 (3.4, 15.1)**
Diverticular disease of intestine	**8.5 (4.6, 12.4)**
Colon polyps	**3.8 (1.8, 5.7)**
Gastritis and duodenitis	4.4 (-1.5, 10.3)
Noninfective enteritis and colitis	-1.2 (-7.1, 4.7)
Inguinal hernia	-5.8 (-11.7, 0.1)
**Joint disorder**	
Osteoarthritis	1.8 (-2.1, 5.7)
**Genitourinary disease**	
Urinary tract infection	**22.7 (14.8, 30.5)**
Kidney stones	4.2 (-5.6, 14.0)
Hyperplasia of prostate	-3.1 (-12.9, 6.7)
Female genital prolapse	-4.5 (-14.3, 5.3)
**Other diseases**	
Diabetes mellitus	**23.0 (17.1, 28.9)**
Iron deficiency anemia	**9.4 (3.5, 15.3)**
Cellulitis	**8.1 (0.3, 15.9)**
Nonmelanoma skin cancer	-2.4 (-6.3, 1.6)
Cataract	3.3 (-0.6, 7.3)
Benign neoplasms of the uterus	7.3 (-0.6, 15.1)
Carpal tunnel syndrome	5.0 (-2.8, 12.8)

HRs and 95% CIs were estimated using Cox regression with age as the underlying time variable. Models were adjusted for age (continuous), sex, self-reported racial/ethnic group (White, other), socioeconomic status (Townsend index, fifths), education level (College or university degree/vocational qualification, further education, school leaver), employment (paid/self-employment, not employment, retired), smoking status (never, previous, current moderate (<15 cigarettes per day), current heavy (≥15 cigarettes per day), current unknown), alcohol consumption frequency (never, <3, 3+ /wk), BMI (<25, 25.0-29.9, 30.0-34.9, 35+ kg/m^2), and for females: HRT use (current, former, never), oral contraceptive pill (current, former, never), menopause status (yes, no), parity (none, 1-2, 3+). Estimates are bold where lower CI > 0.

Abbreviations: CI=confidence interval; HR=hazard ratio; HRT= hormone replacement therapy; MVPA=moderate-to-vigorous physical activity; PAR=population attributable risk.

© 2023 Watts EL et al. *JAMA Network Open*.

eTable 5. Associations of Mean Accelerometer-Measured Physical Activity (Fourths) With Risks of 25 Common Conditions

Condition	Fourth	N cases	Median milligravity units (range)	HR by fourths (95% CI)
Venous thromboembolism	1	241	21 (0.4-24.1)	1 (ref)
	2	186	26.5 (24.1-28.9)	0.93 (0.76, 1.12)
	3	134	31.5 (28.9-34.6)	**0.76 (0.61, 0.94)**
	4	112	39.3 (34.6-83.8)	**0.73 (0.57, 0.92)**
Ischemic stroke	1	160	21.3 (0.4-24.3)	1 (ref)
	2	107	26.8 (24.3-29.2)	0.82 (0.64, 1.05)
	3	91	31.8 (29.2-34.9)	0.81 (0.62, 1.06)
	4	68	39.6 (34.9-83.8)	0.74 (0.55, 1.00)
Ischemic heart disease	1	582	21.3 (0.4-24.3)	1 (ref)
	2	447	26.8 (24.3-29.2)	0.94 (0.83, 1.06)
	3	398	31.8 (29.2-34.9)	0.94 (0.83, 1.08)
	4	315	39.6 (34.9-83.8)	0.86 (0.75, 1.00)
Atrial fibrillation and flutter	1	290	21.3 (0.4-24.3)	1 (ref)
	2	233	26.8 (24.3-29.2)	0.96 (0.81, 1.14)
	3	210	31.8 (29.2-34.9)	0.99 (0.82, 1.18)
	4	165	39.6 (34.9-83.8)	0.92 (0.75, 1.13)
Hemorrhoids	1	276	21 (0.4-24.1)	1 (ref)
	2	274	26.5 (24.1-28.9)	0.96 (0.81, 1.14)
	3	275	31.5 (28.9-34.6)	0.95 (0.80, 1.12)
	4	326	39.3 (34.6-83.8)	1.08 (0.91, 1.29)
Pneumonia	1	388	21 (0.4-24.1)	1 (ref)
	2	218	26.5 (24.1-28.9)	**0.68 (0.58, 0.81)**
	3	162	31.5 (28.9-34.6)	**0.57 (0.48, 0.69)**
	4	173	39.3 (34.6-83.8)	**0.73 (0.60, 0.88)**
Gallbladder disease	1	447	21 (0.4-24.1)	1 (ref)
	2	273	26.6 (24.1-29)	**0.69 (0.59, 0.80)**
	3	220	31.6 (29-34.7)	**0.60 (0.51, 0.71)**
	4	168	39.0 (34.7-83.8)	**0.50 (0.42, 0.61)**
Gastro-esophageal reflux disease	1	274	21.1 (0.4-24.1)	1 (ref)
	2	269	26.6 (24.1-29)	1.02 (0.86, 1.21)
	3	224	31.6 (29-34.7)	0.88 (0.73, 1.05)
	4	202	39.4 (34.7-83.8)	0.83 (0.68, 1.00)
Diverticular disease	1	608	21.1 (0.4-24.1)	1 (ref)
	2	533	26.6 (24.1-29)	0.95 (0.85, 1.07)
	3	530	31.6 (29-34.7)	1.01 (0.90, 1.14)
	4	425	39.4 (34.7-83.8)	0.88 (0.77, 1.01)
Gastritis and duodenitis	1	428	21.1 (0.4-24.1)	1 (ref)
	2	349	26.6 (24.1-29)	0.85 (0.74, 0.99)
	3	347	31.5 (29-34.7)	0.88 (0.76, 1.02)
	4	314	39.3 (34.7-83.8)	**0.83 (0.72, 0.97)**
Colon polyps	1	1603	21.1 (0.4-24.1)	1 (ref)
	2	1453	26.6 (24.1-29)	0.95 (0.88, 1.02)
	3	1477	31.6 (29-34.7)	1.00 (0.93, 1.08)
	4	1317	39.4 (34.7-83.8)	0.94 (0.87, 1.01)
Non-infective enteritis and colitis	1	167	21 (0.4-24.1)	1 (ref)
	2	155	26.5 (24.1-28.9)	0.95 (0.76, 1.18)
	3	156	31.5 (28.9-34.6)	0.98 (0.78, 1.22)
	4	162	39.3 (34.6-83.8)	1.06 (0.84, 1.33)
Inguinal hernia	1	325	21 (0.4-24.1)	1 (ref)
	2	330	26.5 (24.1-28.9)	**1.20 (1.02, 1.40)**
	3	298	31.5 (28.9-34.6)	1.19 (1.01, 1.39)
	4	310	39.3 (34.6-83.8)	**1.34 (1.14, 1.58)**
Osteoarthritis	1	736	21.2 (0.9-24.2)	1 (ref)

© 2023 Watts EL et al. *JAMA Network Open*.

	2	714	26.7 (24.2-29.1)	**1.15 (1.04, 1.28)**
	3	622	31.7 (29.1-34.8)	**1.15 (1.03, 1.28)**
	4	630	39.5 (34.8-83.8)	**1.43 (1.27, 1.60)**
Urinary tract infection	1	238	21 (0.4-24.1)	1 (ref)
	2	148	26.5 (24.1-28.9)	**0.74 (0.60, 0.91)**
	3	118	31.5 (28.9-34.6)	**0.67 (0.53, 0.84)**
	4	81	39.3 (34.6-83.8)	**0.54 (0.41, 0.70)**
Kidney stones	1	100	21 (0.4-24.1)	1 (ref)
	2	77	26.5 (24.1-28.9)	0.82 (0.61, 1.11)
	3	71	31.5 (28.9-34.6)	0.78 (0.57, 1.07)
	4	61	39.3 (34.6-83.8)	**0.67 (0.48, 0.94)**
Enlarged prostate	1	147	20.3 (0.9-23.5)	1 (ref)
	2	146	26 (23.5-28.4)	1.08 (0.85, 1.36)
	3	133	31 (28.4-34.2)	1.06 (0.83, 1.34)
	4	116	39.1 (34.2-81.5)	1.05 (0.82, 1.36)
Female genital prolapse	1	131	21.6 (0.4-24.6)	1 (ref)
	2	140	27.1 (24.6-29.4)	1.13 (0.89, 1.44)
	3	120	32 (29.4-35)	1.02 (0.79, 1.31)
	4	114	39.6 (35.0-83.8)	1.06 (0.81, 1.38)
Diabetes	1	737	21 (0.4-24)	1 (ref)
	2	408	26.5 (24-28.9)	**0.77 (0.68, 0.87)**
	3	328	31.5 (28.9-34.6)	**0.76 (0.66, 0.87)**
	4	205	39.3 (34.6-83.8)	**0.58 (0.50, 0.69)**
Iron deficiency anemia	1	262	21 (0.4-24)	1 (ref)
	2	187	26.5 (24-28.9)	**0.79 (0.65, 0.95)**
	3	197	31.5 (28.9-34.6)	0.89 (0.73, 1.07)
	4	158	39.3 (34.6-83.8)	**0.76 (0.62, 0.94)**
Cellulitis	1	159	21 (0.4-24.1)	1 (ref)
	2	121	26.5 (24.1-28.9)	0.92 (0.72, 1.17)
	3	105	31.5 (28.9-34.6)	0.89 (0.69, 1.15)
	4	85	39.3 (34.6-83.8)	0.83 (0.62, 1.10)
Nonmelanoma skin cancer	1	545	21 (0.4-24.1)	1 (ref)
	2	459	26.6 (24.1-29)	0.96 (0.84, 1.08)
	3	378	31.5 (29-34.6)	0.86 (0.75, 0.99)
	4	352	39.4 (34.6-83.8)	0.93 (0.80, 1.07)
Cataracts	1	1537	21.1 (0.4-24.1)	1 (ref)
	2	1147	26.6 (24.1-29)	**0.88 (0.82, 0.95)**
	3	1017	31.6 (29-34.7)	**0.91 (0.84, 0.99)**
	4	824	39.4 (34.7-83.8)	0.94 (0.86, 1.03)
Benign neoplasms of the uterus	1	152	22 (0.4-25)	1 (ref)
	2	137	27.4 (25-29.7)	0.88 (0.70, 1.11)
	3	142	32.3 (29.7-35.4)	0.90 (0.71, 1.14)
	4	141	39.9 (35.4-81.3)	0.85 (0.67, 1.08)
Carpal tunnel syndrome	1	136	21 (0.4-24)	1 (ref)
	2	134	26.5 (24-28.9)	1.18 (0.93, 1.50)
	3	149	31.4 (28.9-34.5)	**1.49 (1.17, 1.89)**
	4	157	39.2 (34.5-83.8)	**1.85 (1.45, 2.37)**

HRs and 95% CIs were estimated using Cox regression with age as the underlying time variable. Models were stratified by age group (< 50, 50–54, 55–59, 60–64, 65-70, and ≥ 70 years), and sex and adjusted for self-reported racial/ethnic group (White, other), socioeconomic status (Townsend index, fifths), education level (College or university degree/vocational qualification, further education, school leaver), employment (paid/self-employment, not employment, retired), smoking status (never, previous, current moderate (<15 cigarettes per day), current heavy (≥15 cigarettes per day), current unknown), alcohol consumption frequency (never, <3, 3+ /wk), BMI (<25, 25.0-29.9, 30.0-34.9, 35+ kg/m^2), and for females: HRT use (current, former, never), oral contraceptive pill (current, former, never), menopause status (yes, no), parity (none, 1-2, 3+). The boxes represent the HRs, and vertical lines represent 95% CIs. Risk estimates are bold where p<0.03.
Abbreviations: CI=confidence interval; HR=hazard ratio; HRT= hormone replacement therapy.

© 2023 Watts EL et al. *JAMA Network Open*.

eTable 6. Sequential Model Adjustments for Mean Accelerometer-Measured Physical Activity (Milligravity Units) and Risks of 25 Common Conditions

	Model 1		Model 2		Model 3		Model 4	
	HR per 1 SD (95% CI)	P	HR per 1 SD (95% CI)	P	HR per 1 SD (95% CI)	P	HR per 1 SD (95% CI)	P
Circulatory disease								
Venous thromboembolism	**0.74 (0.68, 0.81)**	**<0.0001**	**0.74 (0.68, 0.81)**	**<0.0001**	**0.75 (0.68, 0.82)**	**<0.0001**	**0.75 (0.68, 0.82)**	**<0.0001**
Ischemic stroke	**0.82 (0.73, 0.91)**	**0.0003**	**0.82 (0.74, 0.92)**	**0.001**	**0.83 (0.75, 0.93)**	**0.001**	**0.83 (0.74, 0.93)**	**0.001**
Ischemic heart disease	**0.89 (0.84, 0.94)**	**<0.0001**	**0.89 (0.85, 0.94)**	**<0.0001**	**0.91 (0.86, 0.95)**	**0.0001**	**0.91 (0.86, 0.95)**	**0.0002**
Atrial fibrillation and flutter	0.96 (0.89, 1.03)	0.26	0.96 (0.90, 1.03)	0.29	0.96 (0.89, 1.03)	0.25	0.96 (0.89, 1.03)	0.25
Hemorrhoids	1.03 (0.97, 1.09)	0.38	1.03 (0.97, 1.09)	0.36	1.03 (0.97, 1.09)	0.40	1.03 (0.97, 1.09)	0.38
Respiratory disease								
Pneumonia	**0.79 (0.73, 0.85)**	**<0.0001**	**0.80 (0.74, 0.86)**	**<0.0001**	**0.82 (0.76, 0.88)**	**<0.0001**	**0.82 (0.76, 0.88)**	**<0.0001**
Digestive disease								
Gallbladder disease	**0.65 (0.60, 0.69)**	**<0.0001**	**0.65 (0.61, 0.70)**	**<0.0001**	**0.66 (0.62, 0.71)**	**<0.0001**	**0.66 (0.62, 0.71)**	**<0.0001**
Gastro-esophageal reflux disease	**0.90 (0.84, 0.96)**	**0.002**	**0.90 (0.84, 0.97)**	**0.003**	**0.91 (0.85, 0.97)**	**0.005**	**0.91 (0.85, 0.98)**	**0.007**
Diverticular disease	**0.90 (0.86, 0.95)**	**<0.0001**	**0.91 (0.86, 0.95)**	**<0.0001**	**0.91 (0.86, 0.95)**	**<0.0001**	**0.91 (0.87, 0.95)**	**<0.0001**
Gastritis and duodenitis	0.94 (0.89, 0.99)	0.03	0.95 (0.89, 1.00)	0.05	0.95 (0.90, 1.01)	0.10	0.96 (0.90, 1.01)	0.11
Colon polyps	**0.94 (0.92, 0.97)**	**<0.0001**	**0.94 (0.92, 0.97)**	**<0.0001**	**0.94 (0.92, 0.97)**	**<0.0001**	**0.95 (0.92, 0.97)**	**0.0001**
Non-infective enteritis and colitis	0.98 (0.90, 1.06)	0.58	0.98 (0.90, 1.06)	0.63	0.98 (0.91, 1.07)	0.68	0.99 (0.91, 1.07)	0.75
Inguinal hernia	**1.17 (1.11, 1.24)**	**<0.0001**	**1.17 (1.11, 1.24)**	**<0.0001**	**1.18 (1.11, 1.24)**	**<0.0001**	**1.18 (1.11, 1.24)**	**<0.0001**
Joint disorder								
Osteoarthritis	1.03 (0.99, 1.07)	0.15	1.03 (0.99, 1.07)	0.15	1.03 (0.99, 1.07)	0.14	1.03 (0.99, 1.08)	0.10
Genitourinary disease								
Urinary tract infection	**0.71 (0.65, 0.78)**	**<0.0001**	**0.72 (0.65, 0.79)**	**<0.0001**	**0.73 (0.66, 0.80)**	**<0.0001**	**0.73 (0.66, 0.80)**	**<0.0001**
Kidney stones	**0.85 (0.75, 0.96)**	**0.01**	**0.85 (0.76, 0.96)**	**0.01**	**0.87 (0.77, 0.98)**	**0.02**	**0.87 (0.77, 0.98)**	**0.02**
Enlarged prostate	1.04 (0.95, 1.13)	0.43	1.04 (0.95, 1.13)	0.38	1.04 (0.95, 1.13)	0.44	1.04 (0.95, 1.13)	0.44
Female genital prolapse	1.01 (0.92, 1.10)	0.91	1.00 (0.91, 1.10)	0.95	1.01 (0.92, 1.11)	0.77	1.01 (0.92, 1.11)	0.82
Other diseases								
Diabetes	**0.61 (0.57, 0.65)**	**<0.0001**	**0.62 (0.59, 0.66)**	**<0.0001**	**0.64 (0.61, 0.68)**	**<0.0001**	**0.65 (0.61, 0.68)**	**<0.0001**
Iron deficiency anemia	**0.87 (0.81, 0.94)**	**0.0004**	**0.88 (0.81, 0.94)**	**0.0006**	**0.89 (0.82, 0.96)**	**0.002**	**0.89 (0.82, 0.96)**	**0.002**
Cellulitis	**0.82 (0.74, 0.91)**	**0.0001**	**0.83 (0.75, 0.91)**	**0.0002**	**0.83 (0.75, 0.92)**	**0.0004**	**0.84 (0.76, 0.92)**	**0.0005**
Nonmelanoma skin cancer	0.98 (0.93, 1.03)	0.42	0.98 (0.93, 1.03)	0.50	0.98 (0.93, 1.03)	0.37	0.98 (0.93, 1.03)	0.37
Cataracts	**0.96 (0.93, 0.99)**	**0.01**	**0.96 (0.93, 0.99)**	**0.02**	**0.96 (0.93, 0.99)**	**0.02**	**0.96 (0.93, 1.00)**	**0.03**
Benign lesions of the uterus	**0.90 (0.83, 0.98)**	**0.02**	**0.91 (0.83, 0.99)**	**0.03**	**0.91 (0.83, 0.99)**	**0.03**	0.91 (0.84, 1.00)	0.04
Carpal tunnel syndrome	**1.13 (1.04, 1.23)**	**0.005**	**1.13 (1.04, 1.23)**	**0.003**	**1.14 (1.05, 1.24)**	**0.002**	**1.15 (1.05, 1.25)**	**0.001**

HRs and 95% CIs were estimated using Cox regression with age as the underlying time variable. Risk estimates are bold where p<0.03.
Model 1: Stratified by age group and sex.
Model 2: Model 1 + adjusted for self-reported racial/ethnic group (White, other), socioeconomic status (Townsend index, fifths), education level (College or university degree/vocational qualification, further education, school leaver), employment (paid/self-employment, not employment, retired)
Model 3: Model 2 + adjusted for smoking status (never, previous, current moderate (<15 cigarettes per day), current heavy (≥15 cigarettes per day), current unknown), alcohol consumption frequency (never, <3, 3+ /wk)
Model 4: Model 3 + adjusted for HRT use (current, former, never), oral contraceptive pill (current, former, never), menopause status (yes, no), parity (none, 1-2, 3+)

© 2023 Watts EL et al. *JAMA Network Open*.

	Model 5		Model 6		Model 7		Model 8	
	HR per 1SD (95% CI)	P	HR per 1SD (95% CI)	P	HR per 1SD (95% CI)	P	HR per 1SD (95% CI)	P
Circulatory disease								
Venous thromboembolism	**0.82 (0.75, 0.90)**	**<0.0001**	**0.83 (0.76, 0.91)**	**<0.0001**	**0.83 (0.75, 0.90)**	**<0.0001**	**0.83 (0.76, 0.90)**	**<0.0001**
Ischemic stroke	**0.85 (0.76, 0.95)**	**0.004**	**0.87 (0.78, 0.98)**	**0.02**	**0.88 (0.78, 0.98)**	**0.02**	0.88 (0.79, 0.99)	0.03
Ischemic heart disease	**0.95 (0.90, 1.00)**	**0.04**	0.97 (0.92, 1.02)	0.20	0.97 (0.92, 1.02)	0.25	0.97 (0.92, 1.03)	0.30
Atrial fibrillation and flutter	1.00 (0.92, 1.07)	0.90	1.00 (0.93, 1.08)	0.91	1.01 (0.94, 1.08)	0.86	1.00 (0.93, 1.08)	0.99
Hemorrhoids	1.02 (0.96, 1.09)	0.47	1.04 (0.98, 1.10)	0.25	1.04 (0.98, 1.10)	0.25	1.04 (0.98, 1.10)	0.24
Respiratory disease								
Pneumonia	**0.83 (0.77, 0.89)**	**<0.0001**	**0.87 (0.81, 0.94)**	**0.0003**	**0.87 (0.81, 0.94)**	**0.0004**	**0.87 (0.81, 0.94)**	**0.0005**
Digestive disease								
Gallbladder disease	**0.74 (0.69, 0.79)**	**<0.0001**	**0.76 (0.70, 0.81)**	**<0.0001**	**0.76 (0.70, 0.81)**	**<0.0001**	**0.76 (0.71, 0.82)**	**<0.0001**
Gastro-esophageal reflux disease	**0.92 (0.86, 0.99)**	**0.03**	0.96 (0.89, 1.02)	0.20	0.96 (0.89, 1.03)	0.21	0.96 (0.89, 1.03)	0.25
Diverticular disease	**0.94 (0.90, 0.99)**	**0.02**	0.96 (0.92, 1.01)	0.13	0.97 (0.92, 1.01)	0.15	0.97 (0.92, 1.02)	0.18
Gastritis and duodenitis	0.96 (0.90, 1.01)	0.12	0.99 (0.93, 1.04)	0.63	0.99 (0.93, 1.05)	0.70	0.99 (0.93, 1.05)	0.67
Colon polyps	**0.96 (0.94, 0.99)**	**0.007**	0.98 (0.95, 1.01)	0.18	0.98 (0.95, 1.01)	0.22	0.98 (0.96, 1.01)	0.24
Non-infective enteritis and colitis	0.98 (0.90, 1.07)	0.70	1.01 (0.93, 1.10)	0.86	1.00 (0.92, 1.09)	0.93	1.01 (0.92, 1.10)	0.87
Inguinal hernia	**1.13 (1.07, 1.19)**	**<0.0001**	**1.13 (1.07, 1.20)**	**<0.0001**	**1.13 (1.07, 1.19)**	**<0.0001**	**1.13 (1.06, 1.19)**	**<0.0001**
Joint disorder								
Osteoarthritis	**1.15 (1.10, 1.19)**	**<0.0001**	**1.16 (1.12, 1.21)**	**<0.0001**	**1.16 (1.12, 1.21)**	**<0.0001**	**1.16 (1.11, 1.21)**	**<0.0001**
Genitourinary disease								
Urinary tract infection	**0.76 (0.69, 0.84)**	**<0.0001**	**0.80 (0.73, 0.89)**	**<0.0001**	**0.80 (0.73, 0.89)**	**<0.0001**	**0.80 (0.73, 0.89)**	**<0.0001**
Kidney stones	0.89 (0.79, 1.00)	0.06	0.90 (0.80, 1.02)	0.09	0.90 (0.79, 1.01)	0.08	0.90 (0.79, 1.02)	0.09
Enlarged prostate	1.02 (0.93, 1.12)	0.63	1.02 (0.94, 1.12)	0.60	1.03 (0.94, 1.13)	0.55	1.03 (0.94, 1.13)	0.48
Female genital prolapse	1.04 (0.94, 1.15)	0.43	1.04 (0.95, 1.15)	0.40	1.04 (0.95, 1.15)	0.40	1.04 (0.94, 1.15)	0.46
Other diseases								
Diabetes	**0.79 (0.74, 0.84)**	**<0.0001**	**0.82 (0.78, 0.87)**	**<0.0001**	**0.83 (0.79, 0.88)**	**<0.0001**	**0.84 (0.79, 0.89)**	**<0.0001**
Iron deficiency anemia	**0.91 (0.84, 0.98)**	**0.02**	0.95 (0.88, 1.03)	0.18	0.95 (0.88, 1.03)	0.20	0.96 (0.88, 1.03)	0.26
Cellulitis	0.93 (0.84, 1.03)	0.14	0.96 (0.86, 1.06)	0.40	0.96 (0.86, 1.06)	0.40	0.96 (0.86, 1.06)	0.38
Nonmelanoma skin cancer	0.96 (0.92, 1.02)	0.19	0.97 (0.92, 1.02)	0.23	0.97 (0.92, 1.03)	0.31	0.97 (0.92, 1.03)	0.32
Cataracts	0.98 (0.94, 1.01)	0.17	0.99 (0.96, 1.02)	0.52	0.99 (0.96, 1.02)	0.58	0.99 (0.96, 1.03)	0.66
Benign lesions of the uterus	0.97 (0.89, 1.07)	0.57	0.98 (0.90, 1.07)	0.67	0.98 (0.90, 1.07)	0.66	0.99 (0.90, 1.08)	0.74
Carpal tunnel syndrome	**1.28 (1.18, 1.40)**	**<0.0001**	**1.31 (1.20, 1.43)**	**<0.0001**	**1.31 (1.20, 1.43)**	**<0.0001**	**1.31 (1.20, 1.42)**	**<0.0001**

HRs and 95% CIs were estimated using Cox regression with age as the underlying time variable. Risk estimates are bold where p<0.03.
Model 5: Model 4 + adjusted for BMI (<25, 25-29.9, 30+ kg/m^2)
Model 6: Model 5 + adjusted for attendance/disability/mobility allowance (attendance allowance, disability living allowance, blue badge, none, missing), self-reported health (excellent, good, fair, poor, missing)
Model 7: Model 6 + adjusted for hypertension (yes, no, missing), cholesterol medication (yes, no, missing), blood pressure medication (yes, no, missing)
Model 8: Model 7 + adjusted for red and processed meat consumption (none, 1-3, 3-6 7+ times per week, missing), fruit and vegetable consumption (none, 1-3, 3-6, 7+ servings per day, missing)

© 2023 Watts EL et al. *JAMA Network Open*.

eTable 7. Associations of Accelerometer-Measured Time Spent Sleeping With Risks of 25 Common Conditions

Condition	N cases	HR per 1 SD (95% CI)*	P-value
Circulatory disease			
Venous thromboembolism	673	1.05 (0.98, 1.14)	0.18
Ischemic stroke	426	1.05 (0.95, 1.16)	0.31
Ischemic heart disease	1742	0.98 (0.94, 1.03)	0.49
Atrial fibrillation and flutter	898	0.93 (0.87, 0.99)	0.03
Hemorrhoids	1151	1.00 (0.94, 1.06)	0.91
Respiratory disease			
Pneumonia	941	0.95 (0.89, 1.01)	0.09
Digestive disease			
Gallbladder disease	1108	**1.08 (1.02, 1.14)**	**0.01**
Gastro-esophageal reflux disease	969	1.06 (0.99, 1.13)	0.07
Diverticular disease	2096	1.02 (0.98, 1.07)	0.31
Gastritis and duodenitis	1438	**1.06 (1.01, 1.12)**	**0.02**
Colon polyps	5850	1.01 (0.98, 1.03)	0.70
Non-infective enteritis and colitis	640	1.04 (0.96, 1.13)	0.32
Inguinal hernia	1263	**0.93 (0.88, 0.98)**	**0.008**
Joint disorder			
Osteoarthritis	2702	**0.95 (0.91, 0.99)**	**0.007**
Genitourinary disease			
Urinary tract infection	585	1.05 (0.97, 1.14)	0.22
Kidney stones	309	1.07 (0.95, 1.19)	0.26
Enlarged prostate	542	0.94 (0.87, 1.03)	0.17
Female genital prolapse	505	1.02 (0.93, 1.12)	0.70
Other diseases			
Diabetes	1678	0.99 (0.94, 1.03)	0.62
Iron deficiency anemia	804	**0.91 (0.85, 0.97)**	**0.005**
Cellulitis	470	**0.89 (0.81, 0.97)**	**0.01**
Nonmelanoma skin cancer	1734	1.04 (0.99, 1.09)	0.11
Cataracts	4525	0.99 (0.96, 1.02)	0.54
Benign neoplasms of the uterus	572	0.99 (0.92, 1.08)	0.91
Carpal tunnel syndrome	576	**0.88 (0.81, 0.95)**	**0.001**

*SD = 1.26 hrs

HRs and 95% CIs were estimated using Cox regression with age as the underlying time variable. Models were stratified by age group (< 50, 50–54, 55–59, 60–64, 65-70, and ≥ 70 years), and sex and adjusted for self-reported racial/ethnic group (White, other), socioeconomic status (Townsend index, fifths), education level (College or university degree/vocational qualification, further education, school leaver), employment (paid/self-employment, not employment, retired), smoking status (never, previous, current moderate (<15 cigarettes per day), current heavy (≥15 cigarettes per day), current unknown), alcohol consumption frequency (never, <3, 3+ /wk), BMI (<25, 25.0-29.9, 30.0-34.9, 35+ kg/m^2), and for females: HRT use (current, former, never), oral contraceptive pill (current, former, never), menopause status (yes, no), parity (none, 1-2, 3+). Risk estimates are bold where p<0.03.

Abbreviations: CI=confidence interval; HR=hazard ratio; HRT= hormone replacement therapy.

© 2023 Watts EL et al. *JAMA Network Open*.

eReferences

1. Craig CL, Marshall AL, Sjöström M, et al. International physical activity questionnaire: 12-country reliability and validity. *Med Sci Sports Exerc*. Aug 2003;35(8):1381-95. doi:10.1249/01.Mss.0000078924.61453.Fb
2. Herbert A, Wijlaars L, Zylbersztejn A, Cromwell D, Hardelid P. Data Resource Profile: Hospital Episode Statistics Admitted Patient Care (HES APC). *Int J Epidemiol*. Aug 1 2017;46(4):1093-1093i. doi:10.1093/ije/dyx015
3. Digital N. Hospital Admitted Patient Care Activity 2018-19. https://digital.nhs.uk/data-and-information/publications/statistical/hospital-admitted-patient-care-activity/2018-19
4. Saint-Maurice PF, Troiano RP, Bassett DR, Jr, et al. Association of Daily Step Count and Step Intensity With Mortality Among US Adults. *JAMA*. 2020;323(12):1151-1160. doi:10.1001/jama.2020.1382
5. Graubard BI, Flegal KM, Williamson DF, Gail MH. Estimation of attributable number of deaths and standard errors from simple and complex sampled cohorts. *Stat Med*. Jun 15 2007;26(13):2639-49. doi:10.1002/sim.2734

© 2023 Watts EL et al. *JAMA Network Open*.
