# Supplementary material for: Association of Accelerometer-Measured Physical Activity Level With Risks of Hospitalization for 25 Common Health Conditions in UK Adults
Source: JAMA Netw Open. 2023 Feb 16;6(2):e2256186. doi: 10.1001/jamanetworkopen.2022.56186 (PMC9936337; doi:10.1001/jamanetworkopen.2022.56186)
Supplement: Supplement 2. — Data Sharing Statement [file jamanetwopen-e2256186-s002.pdf]

Data Sharing Statement

Watts. Association of Accelerometer-Measured Physical Activity Level With Risks of Hospitalization for 25 Common Health Conditions in UK Adults. *JAMA Netw Open*. Published February 16, 2023. doi:10.1001/jamanetworkopen.2022.56186

Data
Data available: No

Additional Information
Explanation for why data not available: We do have permission to share the data. However, all bona fide researchers can apply to use the UK Biobank resource for health-related research that is in the public interest (https://www.ukbiobank.ac.uk/register-apply/).
